# Supplementary material for: The journey through care: study protocol for a longitudinal qualitative interview study to investigate the healthcare experiences and preferences of children and young people with life-limiting and life-threatening conditions and their families in the West Midlands, UK
Source: BMJ Open. 2018 Jan 21;8(1):e018266. doi: 10.1136/bmjopen-2017-018266 (PMC5780714; doi:10.1136/bmjopen-2017-018266)
Supplement: Author's manuscript [file bmjopen-2017-018266.draft_revisions.pdf]

BMJ Open

BMJ Open is committed to open peer review. As part of this commitment we make the peer review history of every article we publish publicly available.

When an article is published we post the peer reviewers' comments and the authors' responses online. We also post the versions of the paper that were used during peer review. These are the versions that the peer review comments apply to.

The versions of the paper that follow are the versions that were submitted during the peer review process. They are not the versions of record or the final published versions. They should not be cited or distributed as the published version of this manuscript.

BMJ Open is an open access journal and the full, final, typeset and author-corrected version of record of the manuscript is available on our site with no access controls, subscription charges or pay-per-view fees (http://bmjopen.bmj.com).

If you have any questions on BMJ Open's open peer review process please email editorial.bmjopen@bmj.com

The Journey through Care: A longitudinal qualitative interview study to investigate the healthcare experiences and preferences of children and young people with life-limiting and life-threatening conditions, and their families.

Journal:	*BMJ Open*
Manuscript ID	bmjopen-2017-018266
Article Type:	Protocol
Date Submitted by the Author:	16-Jun-2017
Complete List of Authors:	Mitchell, Sarah; Warwick Medical School<br>Slowther, Anne-Marie; University of Warwick, Warwick Medical School<br>Coad, Jane; Coventry University<br>Dale, Jeremy; University of Warwick, Warwick Medical School
**Primary Subject Heading**:	Palliative care
Secondary Subject Heading:	Health services research, Paediatrics, Patient-centred medicine, Qualitative research
Keywords:	Organisation of health services < HEALTH SERVICES ADMINISTRATION & MANAGEMENT, Paediatric palliative care < PAEDIATRICS, PALLIATIVE CARE, QUALITATIVE RESEARCH

SCHOLARONE™ Manuscripts

For peer review only

The Journey through Care: A longitudinal qualitative interview study to investigate the healthcare experiences and preferences of children and young people with life-limiting and life-threatening conditions, and their families

Mitchell S[1], Slowther A-M[1], Coad J[2], Dale J[1].

Corresponding Author: Sarah Mitchell MB ChB FRCGP MSc

National Institute of Health Research Doctoral Research Fellow

Sarah.j.mitchell@warwick.ac.uk; 07950202240; @MacGPSarah

Author affiliations:

1. Warwick Medical School, University of Warwick, Gibbet Hill Road, Coventry, CV4 7AL
2. Faculty of Health and Life Sciences, Coventry University, Priory Street, Coventry, CV1 5FB.

Word counts

- Abstract: 352
- Protocol: 3997

For peer review only

Abstract

INTRODUCTION

The numbers of children and young people living with life-limiting and life-threatening conditions is

rising. Providing high quality, responsive healthcare for them and for their families presents a significant challenge. Their conditions are often complex and highly unpredictable. Palliative care is advocated for people with life-limiting and life-threatening conditions, but these services for children are highly variable in terms of availability and scope. Furthermore, the term "palliative care" frequently carries negative connotations and is used inconsistently in policy and practice, providing a barrier to the delivery of such care. Little is known about the lived experiences and preferences of children and their families in terms of the palliative care that they do, or do not, receive.

The aim is to provide an in-depth insight into the experiences and preferences in relation to healthcare services (whether or not this includes a specific palliative care service), including perceptions of the term "palliative care".

METHODS AND ANALYSIS

A qualitative study comprising longitudinal interviews with children (aged 5-18 years) living with life-limiting or life-threatening conditions, their family members.

Data analysis will start with thematic analysis, to be followed by narrative and cross-case analysis to examine changing experiences and preferences over time, at the family level and within the wider healthcare system.

Patient and Public Involvement (PPI) has informed the design and conduct of the study. Findings will be used to develop recommendations for an integrated model of palliative care for CYP in partnership with the PPI group.

ETHICS AND DISSEMINATION

Ethical approval was granted in September 2016 by the NHS Health Research Authority (IRAS ID: 196816, REC reference: 16/WM/0272).

Findings will be of immediate relevance to health care policy makers, commissioners and voluntary sector organisations. Reports will be prepared for these audiences, as well as for children and their families, alongside academic outputs.

STRENGTHS AND LIMITATIONS

- An in-depth, contextual, longitudinal qualitative study with multiple child and family member stories captured over time.
- New insights will be provided because all of the children and families included in the study could benefit from palliative care as it is currently defined, however not all will have had conversations about this or have been referred to specialist palliative care services.

For peer review only

- A diverse study population in terms of age, clinical condition, cultural background and family structure, allowing detailed consideration of the role of healthcare services in effectively recognising and supporting children and families with their individual needs. However all will speak English.
- Neonates, pre-school children and young people at transition (over the age of 18 years) are all excluded and warrant research in their own right.
- There are multiple potential sources of bias which will be addressed throughout the study, including recruitment bias and the unconscious bias of the researcher.

Author's contributions

SM drafted the protocol with regular academic supervision from JD, A-M S and JC. The study was conceptualised by SM, JD, A-MS and JC, informed and guided by Patient and Public Involvement. The protocol incorporates peer review feedback received during the application process for the NIHR Doctoral Research Fellowship, and the MPhil to PhD upgrade panel at the University of Warwick. JD, A-MS and JC have all reviewed the protocol for intellectual content. All authors have reviewed and agreed this version.

Funding statement

This work is supported by a National Institute of Health Research Doctoral Research Fellowship (Dr Sarah Mitchell DRF-2014-07-065)

Competing interests

The authors have no competing interests.

Introduction

Children and young people with life-limiting conditions and life-threatening conditions represent a growing concern in healthcare (1). With advances in clinical practice, the numbers of children living with these conditions is rising (1-3). The nature of their conditions is complex and unpredictable; the

For peer review only

risk of a sudden deterioration and death is an everyday reality for many. Family carers often experience enormous emotional, physical and financial pressures (4).

Research suggests that families of children with life-limiting and life-threatening conditions wish for continuous, holistic healthcare, with the option that this is delivered in the home environment (4). However most children who die have a pre-existing life-limiting condition (5), most die in hospital (6, 7), most frequently in an intensive care environment, where the mode of death is often withdrawal or limitation of life-sustaining treatments (8-10). The length of stay in the intensive care unit before death is increasing and the costs of hospital care at the end of life are significant (11, 12). This situation presents complex clinical and ethical dilemmas at both individual and organisational levels, and an issue relevant for wider societal debate.

For clarity in this paper, children and young people will be referred to throughout as "children".

WHAT IS PALLIATIVE CARE?

Palliative care is an approach to care that is advocated for all people who live with a life-limiting or life-threatening condition. Current definitions of palliative care are broad, which can cause difficulties for those designing and commissioning specific services. Palliative care for children with life-threatening conditions is defined by the World Health Organisation as "a special, albeit closely related field to adult palliative care; the principles apply to other paediatric chronic disorders:

- The active total care of the child's body, mind and spirit, and support to the family.
- It begins when illness is diagnosed, and continues regardless of whether or not a child receives treatment directed at the disease.
- Health providers must evaluate and alleviate a child's physical, psychological, and social distress.
- Effective palliative care requires a broad multidisciplinary approach that includes the family and makes use of available community resources; it can be successfully implemented even if resources are limited.
- It can be provided in tertiary care facilities, in community health centres and even in children's homes (13)."

The UK national charity for paediatric palliative care, Together for Short Lives, defines palliative care for children with life-limiting conditions as "an active and total approach to care, from the point of diagnosis or recognition, embracing physical, emotional, social and spiritual elements through to death and beyond. It focuses on enhancement of quality of life for the child and support for the family and includes the management of distressing symptoms, provision of short breaks and care through death and bereavement" (14).

For peer review only - http://bmjopen.bmj.com/site/about/guidelines.xhtml

WHICH CHILDREN?

The following four categories describing the types of clinical conditions where palliative care could be beneficial have been outlined, as follows (14):

- *Group 1:* life-threatening conditions where access to palliative care services is necessary alongside attempts at curative treatment and / or if treatment fails, such as cancer.
- *Group 2:* conditions such as Duchenne muscular dystrophy, where premature death is inevitable, but where there may be long periods where the child is well.
- *Group 3:* progressive conditions without curative treatment options, such as Batten disease.
- *Group 4:* irreversible but non-progressive conditions, with complex disabilities and healthcare needs which lead to increased likelihood of premature death, such as severe brain injury.

For peer review only

ORGANISATIONAL ISSUES

Currently there is wide geographic variation in terms of paediatric palliative care services, and a poorly understood range of commissioning arrangements to support these services. Many services exist as a result of significant contributions from the voluntary sector (including children's hospices), through the efforts of motivated individuals, and through non-recurring funding opportunities rather than the implementation of national policy (15). A significant development is the emergence of paediatric palliative medicine as a sub-specialty of paediatrics (16, 17).

Effective palliative care services for children require strong partnerships between providers, and may require cross-boundary, collaborative commissioning between the statutory and voluntary sectors (18). In the UK, palliative care for children has specifically been included in national policy , a service specification for paediatric palliative care exists and NICE (National Institute for Health and Care Excellence) Guidelines for End-of-Life Care for Infants, Children and Young People were published in 2016 (19-23).

RATIONALE FOR RESEARCH

Despite the range of recommendations for the provision and development of paediatric palliative care services, there remains a lack of research evidence to support the implementation of these guidelines (1, 17, 24-26). The proposed study is designed to address this gap by exploring the strengths and limitations of current healthcare service provision and the expectations and experiences of those who could potentially benefit from this approach to care.

RESEARCH QUESTIONS

1. How do children with life-limiting and life-threatening conditions and their family members perceive healthcare services, and in particular "palliative care"?
2. What are the experiences and preferences of children living with a life-limiting or life-threatening condition, and/or their families, in relation to the delivery of healthcare services?
3. What are the facilitators and barriers to the delivery of palliative care for children, and how might these be overcome?
4. What should an integrated model of palliative care for children look like?

Methods and Analysis

METHODOLOGY

This study is designed to enhance understanding of the relationships between policy implementation, service design and delivery, and patient and family experiences, and the influences they have on each other; a "causal" intellectual puzzle (27, 28).

The study takes a positivist approach; that reality can be known through a process of observation, identification of regularities and inductive reasoning (29). It draws upon the philosophical models of realism and constructionism which dictate that reality exists and is actively "constructed" by human beings through actions in a social context, rather than being passively received by them (29). By eliciting the views of children and members of their family regarding healthcare services, a detailed meta-synthesis will be produced which will provide valuable insights into that reality, and will then be used to inform recommendations for a model of care for future palliative care services. Since these children and their families often receive care from a range of organisations in both the community and in hospital, in-depth exploration of how services have been accessed over time, and the overall experience of care from a range of providers is possible.

The research plan has been informed by review of relevant literature, patient and public involvement (PPI) work and advice from local experts via the West Midlands Paediatric Palliative Care Network.

METHODS

An in-depth longitudinal qualitative study using semi-structured interviews with school-aged children (5-18 years) and one or two of their household family members.

For peer review only - http://bmjopen.bmj.com/site/about/guidelines.xhtml

Qualitative research methods are suited to complex, emotional subjects to elicit detailed, in-depth insights (30, 31). Benefits of longitudinal studies include being able to describe the changing needs of the CYP, and their experience of services, over time (32), and enabling rapport to build between researcher and participant.

SAMPLING AND RECRUITMENT

Recruitment to a study of this nature depends on many factors, including the clinical condition of the child, conflicting demands on the family's time, and the motivation and understanding of their clinical teams. Recruitment began following ethical approval in October 2016 and will continue until January 2018.

The approach to participants will be through:

1. direct invitation via their clinical team
2. leaflets and posters displayed in public areas in the hospital (such as notice boards on wards and in outpatients).

The research will be introduced to clinical teams in both the hospital and the community through formal presentations at departmental meetings and to individual clinicians at their request, as well as to the paediatric palliative care network. The researcher, SM, will undertake a period of shadowing with clinical teams, on hospital wards, in outpatient clinics and in the community.

Potential participants will be provided with a participant information sheet, with details of the researcher, the project, how to get involved, and a contact telephone number and email address.

Inclusion and exclusion criteria are outlined in Table 1. Our aim initially is to purposively sample children so that each of the four Together for Short Lives categories are represented (14). However, since children live with such individual and highly complex conditions we anticipate that achieving this may be difficult. The study population will therefore be children with life-limiting or life-threatening conditions, aged from 5-18 years, and their family members, some of whom have experience of a palliative care service, and some who do not.

The study has been carefully designed to ensure that all of the children have the opportunity to participate and that wherever possible the views of the child are included, by tailoring each individual interview to their needs and capabilities (including the consent and agreement process). This may include having a learning disability or communication difficulties associated with their condition.

For peer review only

Table 1: Inclusion and Exclusion Criteria

Inclusion Criteria	1. Children aged 5-18 yrs (school age) with a life-limiting or life-threatening condition who are under the care of the Community Children's Nursing Team and/or the Children's Hospital and who either:<br>• receive palliative care services<br>• are aware of (have had discussions about) palliative care services<br>• are living with relapsing or refractory disease<br>• or have had a life-threatening episode (admission to the Paediatric Intensive Care Unit (PICU))<br>2. Their family members, who live in the same household (33).
Exclusion Criteria	• Children aged < 5 years and > 18 years.<br>• Families of children < 5 years and > 18 years old<br>• Children and families with whom I have clinical contact<br>• Children and / or families who do not wish to participate.<br>• Children who are too unwell will not be approached for interview, but their family members may still participate if they wish to.<br>• Children who are unable to participate in a conversational interview for any reason related to their condition will not be approached for interview, but their family members may participate if they wish to.<br>• Children and families who are unable to provide informed consent in English will not be approached for interview.

INTERVIEW PLAN

Interviews will be carried out by SM, a general practitioner with advanced communication skills training and previous experience in qualitative interviewing. According to the preference of participants, interviews will be conducted with an individual or with the child and parent together, in their preferred location. Interviews will be open and conversational, using a blended approach of interview techniques, with passive interviewing allowing the participant space and time to tell their story (narrative), and more active techniques, including appreciative inquiry, which asks "what works well?" and "why does it work well?" (4, 34), employed. A topic guide (table 2) will guide the interview; this will continue to develop iteratively throughout the research, with adaptations made during each interview and in response to each individual participant.

For peer review only - http://bmjopen.bmj.com/site/about/guidelines.xhtml

Table 2: Topic Guide

For all families	For those aware of "palliative care"
**Introduction**<br><br>**Please tell me your story, in any way that you can / want to**<br><br>**Please tell me the story of you**<br><br>*Your Story*<br><br>**Please can you tell me a about you?**<br><br>**Your family?**<br><br>**Your child(ren)**<br><br>**What is important to you?**<br><br>**What do you like to do?**<br><br>**Which places are important to you?**<br><br>**Where do you spend your time?**<br><br>**Which services are involved in your care?**<br><br>**Who comes to see you?**<br><br>**What do they do?**<br><br>What's helpful?<br><br>What's not?<br><br>**Which healthcare professionals do you consider to be key in the delivery of your care?**<br><br>**What works best?**<br><br>Which services / professionals are most	*Palliative care and you (if appriopriate)*<br><br>**Do you have "palliative care" services?**<br><br>Have you ever heard the term "palliative care"?<br><br>What does that mean to you?<br><br>What do you receive those services for?<br>What do these services provide for you?<br>Does it matter what a service is called?<br><br>Do you receive services from the hospice?<br><br>**Can you tell me how you came to receive palliative care / know the palliative care nursing team / the hospice?**<br><br>When were you referred?<br><br>Who brought it up / made the referral?<br><br>How was this discussed with you?<br><br>How was that for you / your family?<br><br>**Do you think that medical / nursing staff receive enough training in this area?**<br><br>What makes you think that?<br><br>**Anything else?**

For peer review only

helpful?<br><br>Which services / professionals do you value most?<br><br>**What doesn't work?**<br><br>**How do you think services could be improved?**<br><br>**Do you talk to other children / young people / families about your healthcare / services"?**<br><br>What do you tell your friends?<br><br>What tends to come up in these discussions?<br><br>Would you recommend these services to others?	

For interviews with children, a range of techniques will be used including de-personalising questions, developing a narrative in the third person, and using props and toys to encourage story-telling. Crafts activities will be used, where appropriate, as a mutual point of focus for the researcher and participant, or as a focus of the interview, as in the draw-write-tell technique (35). PPI advice has been sought on the format of interviews for CYP (Table 3).

Table 3: Feedback from PPI groups on interview plans

January 2016	*"Those who are passionate about improving palliative care will take part regardless of how sensitive this may be"*
July 2016	*"Remember young people who are seriously ill are more mature, they have to grow up"*<br><br>*"Keep it simple as often a child will openly speak anyway"*<br><br>*"'Do you talk about it to your friends?' is a good question, a good way to talk to most ages."*<br><br>*"Children are more eloquent, mature and more capable than you think"*

For peer review only

October 2016	*"Use pictures and images, more emojis"*
February 2017	*"Doesn't make me uncomfortable as I think it is very important and relevant"*

Each interview will be audio-recorded, with field notes to include any additional comments from the child or family made once the audio-recording has stopped (36), reflections on the interview and observation of the family situation, environment, behaviour, and any other interactions that may take place (for example with other members of the family and clinical staff in hospital or on the phone).

Participants will be asked whether they would like to participate in a series of interviews (longitudinal interviews). These will allow the identification of common themes over time and for theories generated through analysis of earlier interviews to be tested out during later interviews. The aim is to continue to conduct interviews until data saturation is achieved (37), however given the uniqueness and individuality of the stories of children and families, it is possible that new themes will continue to emerge such that data saturation is impossible. We will aim for saturation of the main themes that emerge from the data, and identify emergent themes, which may form the basis of future research. Another benefit of longitudinal interviews is the ability to build rapport, particularly with children who may be unsure of the researcher at first.

The time intervals between interviews will be individually agreed, depending on the child and family circumstances. The method of communication with each family will also be individually agreed (phone or email). Up to three interviews with each participant are aimed for.

We anticipate practical challenges with conducting longitudinal interviews relating to fluctuations and changes in the clinical condition of each child. Depending on their condition, some children will respond well to treatments and get better. Others may suffer unexpected complications of their condition or treatment, and some may suffer deteriorations which bring about the possibility of dying. To manage the research in this context, we will check the family understanding of the situation before every interview. On occasions, interviews may need to be postponed and rearranged at late notice due to a change in circumstances.

For children who are unable to participate in interviews due to their condition, family members will be interviewed. Children and families are under no obligation to take part in follow-up interviews if they do not wish to. In these cases, and with their consent, data from previous interviews will still be included in the study.

For peer review only

DATA ANALYSIS

Interviews will be transcribed verbatim, and NVivo used for data handling. All data will be coded, and codes grouped into broad overarching themes. Matrices will be developed to identify key times for families and comparison of the themes identified at these times, for example the time of diagnosis, an admission to intensive care or referral to a palliative care team. This initial analysis will be followed by an in-depth, narrative analysis, using structure-form analysis to examine not just what is being said, but how it is being said, and to position narratives in the context of palliative care from a micro (immediate clinical team), meso (local organisation) and macro (wider healthcare system) level perspective (38, 39).

Peer review and respondent validation will take place throughout the data analysis as follows (40, 41):

1. Peer review: SM will code all of the data. A selection of transcripts will be reviewed and independently coded by other members of the research team in order to decrease lone researcher bias (40). The coding frameworks will be discussed and compared, allowing further development of categories and themes.
2. Respondent validation: By returning to participants to conduct longitudinal interviews, there is opportunity to check, validate or refute emergent themes from the initial data analysis.

Initial findings will be presented to expert groups from different regions of the UK. There are 12 paediatric palliative care networks in the UK, which include professionals from a range of organisations within paediatric palliative care. Several have patient and family representatives. Arrangements will be made to present initial study findings and work in progress to four of the UK networks at existing meetings. An expression of interest email will be circulated to network chairs via Together for Short Lives, and arrangements made to attend meetings from networks who respond. Audio-recorded focus group discussions will be carried out at those meetings by SM.

PATIENT AND PUBLIC INVOLVEMENT

PPI has been integral to the design and conduct of the study. Young people aged 12-22 years who are members of existing groups at a children's hospital and children's hospice have provided advice on the study proposal and design. Smaller groups have been recruited for specific activities, including conference presentations. Group members range in age from 12 to 20 years. PPI will continue throughout the project, with the aim of co-producing the recommendations for the model of care. This will involve structured group sessions during which anonymised findings of the data

For peer review only

analysis will be presented to the group for feedback and comment. A patient experience framework will be used to structure the discussion and to build recommendations (42).

Table 4: PPI activities

Completed PPI activities	• Developing the original research proposal<br>• Advising on the language used in the study (suggesting a change in the title from "Palliative Care for Children and Young People: What? When? How?" to "The Journey through Care".<br>• Developing participant resources including leaflets for older and younger children<br>• Interview design, including suggesting how questions<br>• Providing family perspectives to a literature review, and becoming a co-author on the paper (17).<br>• Taking part in oral presentations at regional conferences
Work in progress	• Designing conference posters and presentations for national conferences<br>• Working as co-researchers to carry out a survey study to investigate understanding of the term "palliative care" for children and young people, and healthcare professionals
Future plans	• Working with a project-specific group to explore the findings of the research study and develop recommendations for a new model of care.<br>• Dissemination projects including conference presentations, posters, website design, use of social media, infographics and films.

STRENGTHS AND LIMITATIONS

The strength of this study lies in the in-depth, contextual qualitative nature of the data, with multiple child and family member stories captured over time. Our anticipated study population is diverse in terms of age, clinical condition, cultural background and family structure, allowing detailed consideration of the role of healthcare services in effectively recognising and supporting children and families with their individual needs. All of the children and families included in the study could benefit from palliative care as it is currently defined (14), however not all will have had conversations about this with their clinicians, or been referred to specialist palliative care services. Potential limitations in the study include our exclusion of neonates, pre-school children and young people at transition (over the age of 18 years). These groups all warrant research in their own right. Given the time and resource constraints of the study, all interviews will be carried out in English. Further research into the experiences of CYP and families who cannot communicate in English is

For peer review only - http://bmjopen.bmj.com/site/about/guidelines.xhtml

necessary. There will be ongoing consideration of sources of bias. Recruitment bias is being addressed by aiming for a diverse sample and providing access to project information independent of the clinical teams. Data saturation will be sought during data analysis, with an ongoing process of reflection and peer review to address any possible unconscious bias of the researcher (SM).

Ethics and Dissemination

Research with children raises ethical and legal considerations around recruitment, consent, and data collection (43, 44). In addition research regarding palliative and end-of-life care can be emotionally demanding and distressing for those involved. There are also particular ethical issues to consider given the longitudinal nature of the study (45).

We are recruiting children and families who are potentially vulnerable and may be experiencing considerable distress. The justification for our approach is that children and their families in this situation are rarely asked about their experiences, but talking to them and understanding their experiences is essential in order to be able to design and develop services that respond to their actual needs. Here we summarise our approach to the ethical issues the study raises.

Figure 1: Ethical issues in longitudinal qualitative research for children and families in palliative care

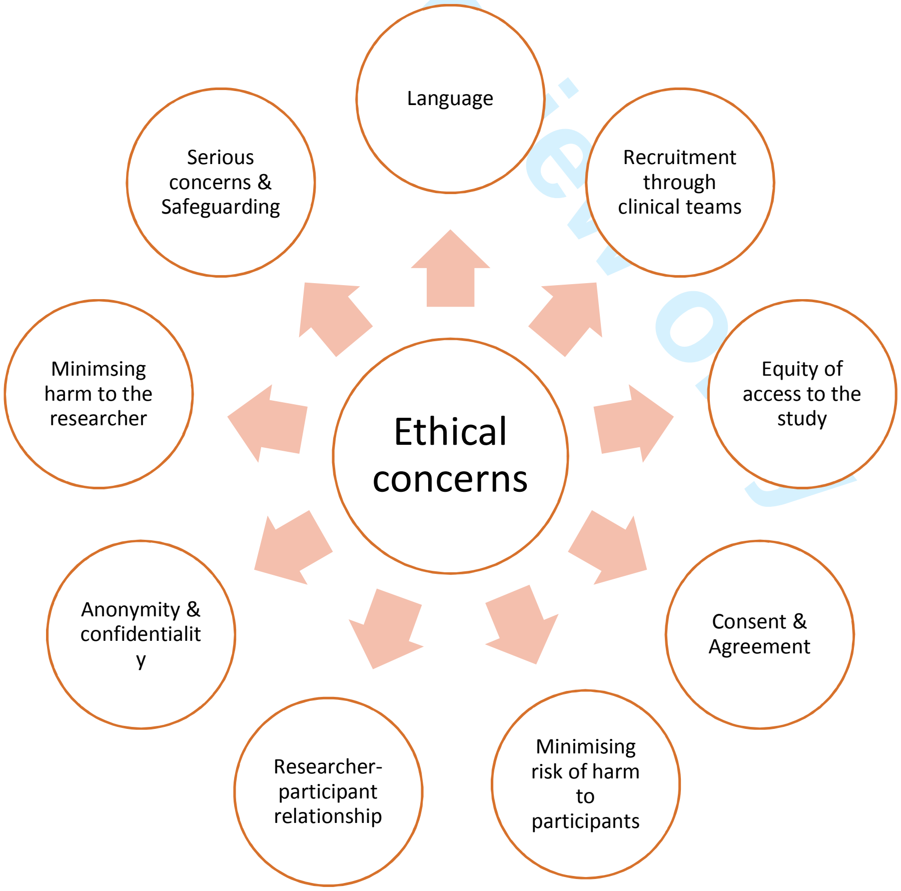

LANGUAGE

Published literature suggests that the term “palliative care” is poorly understood and perceived negatively (46-50), a view confirmed by our PPI group. The scope of our study is therefore to investigate the experiences of children with “life-limiting”, “life-threatening” and “conditions which may or may not get better”, whether or not they have heard of palliative care or receive care from specialist services. “Palliative care” will be avoided in participant information sheets and interviews, unless individuals are already familiar with palliative care services or bring it up themselves.

RECRUITMENT

There is an ethical challenge in terms of potential coercion to the study by clinicians who know the family well. Clinicians will therefore only provide study information but will not actively recruit families; the initial expression of interest is from the family to the researcher. The researcher (SM) will then discuss the study in person or by phone with the child and their family member(s) and answer any questions before arranging a time for interview. Participants will be made aware that they can decline to take part or to withdraw at any stage without having to give a reason. Interviews are only carried out at a time that is mutually agreed and minimises any potential inconvenience or intrusion.

Equity of access to the study

Recruitment through clinical teams is widely used in palliative care research but may be limited by “gatekeeping” (51). There may also be families who wish to participate who do not find out about the study through their clinical team. In order to address this, we have designed posters for display on hospital wards and in outpatients, and at the local children’s hospice, and a paragraph for organisational newsletters. These provide the direct contact details for the researcher (email, text or phone).

The study setting is Birmingham, UK, a city where the population is highly diverse in terms of family situation and multiculturalism. Over 50% of families with a child known to palliative care services in Birmingham and Solihull are from black or minority ethnic backgrounds (52). Many of these families speak English as a first or second language , so within the time and resource constraints of this study, interviews will be carried out with those who can provide informed consent and take part in an interview in English.

CONSENT

Consent for the study raises ethical and legal issues with children who are under the age of 16 years and / or do not have the capacity to consent. We will aim for written and / or verbal consent and agreement from every individual for every interview.

For peer review only

For children under the age of 16, written consent will be obtained from the parent and then verbal or written agreement obtained from the child.

In keeping with the Mental Capacity Act, there is an assumption of capacity in young people aged 16 years and over, so they will be asked for consent first, followed by agreement from their parent(s). Parental agreement is not a legal requirement, but conducting an interview with a young person about a potentially difficult subject without the knowledge or agreement of their parents is an ethical concern. If there is a concern that the young person lacks capacity or is considered particularly vulnerable, for example with a learning disability, parents will be asked to provide verbal and written consent in addition to the young person's agreement.

Parental consent is required for any interview to be carried out in the family home (44).

For a child on a full care order, social worker consent would replace that of parental consent. Where possible parental consent / agreement will also be sought.

INTERVIEWS

Subject areas discussed during interviews may cause distress to participants, and recruitment may occur soon after sensitive conversations. We have designed the study to ensure that the risks and burden associated with taking part in the study are minimal.

Qualitative interviews can be informal and reflexive to the needs of the participant. In the event that a participant experiences any difficulties during the interview, such as tiredness or distress, the interview will be halted, and if necessary brought to an end. Adequate time will be given for debrief, and the researcher will provide information about local resources for support if necessary. Interviews will be carried out at a time and in a location that is convenient to the individual participant. If this is in hospital, the researcher (SM) will liaise closely with clinical teams so that the research does not interfere with routine clinical care and ward work.

Longitudinal interviews

Family views and understanding of what might happen next as a result of the condition of the child will be discussed sensitively, and any follow-up interviews scheduled around possible further treatments. If it seems likely that there will be a deterioration in the condition of the child, this is explored carefully and an agreement made with the individual family about whether they want to continue to participate in the study.

ANONYMITY AND CONFIDENTIALITY

All interview data will be anonymised with personal identifiers removed. Any qualitative interview data that could identify child, families or any professionals involved in their care because of the

For peer review only

individuality and context of the narrative is included in the data analysis, but will be excluded from reporting.

Field notes and anonymised interview transcripts will be stored securely on a password protected university hard drive.

MINIMISING HARM TO THE RESEARCHER

There is a need for clearly defined boundaries for a researcher – participant relationship in a longitudinal study of this type. It will be made clear to participants at the time of consent that it is not the role of the researcher to provide personal support or clinical advice. With the risk of emotional distress for the researcher, plans to ensure adequate support through regular academic supervision and access to a counsellor are in place.

SERIOUS CONCERNS AND SAFEGUARDING

If information contained in a participant's response indicates a serious clinical or safeguarding concern or an issue which may jeopardise the safety of the participant or another person, this will be escalated appropriately in line with the protocols of the community or hospital trusts. This may on very rare occasions necessitate a breach of participant confidentiality in order to maintain their safety. Participants will be informed of any disclosure and to whom it is made.

ETHICAL APPROVAL

Ethical approval was granted by the UK Health Research Authority on 14th September 2016 (IRAS ID: 196816, REC reference: 16/WM/0272, Sponsor: University of Warwick).

PLANNED OUTPUTS AND PLANS FOR IMPACT

The motivation for this research is driven by the clinical experience of the researcher, and the research embedded in plans for impact. Table 5 outlines our planned outputs. We will work on traditional academic and clinical outputs, but simultaneously work with the PPI group to plan innovative, accessible outputs for patients, the public and commissioners which will include infographics and film based reports outlining our recommendations.

Table 5: Planned outputs from the research

Academic / Clinical audiences	Patient, public and policy-maker audiences
• PhD Thesis<br>• Peer reviewed publications<br>• Presentations at national and international conferences	• A report prepared for participants and PPI volunteers<br>• The development of guidance for commissioners and providers

For peer review only - http://bmjopen.bmj.com/site/about/guidelines.xhtml

	• The development of resources that are accessible to patients and families

For peer review only

References

1. Fraser LK, Miller M, Hain R, et al. Rising national prevalence of life-limiting conditions in children in England. Pediatr. 2012;129(4):e923-9.

2. Fraser LK, Parslow RC, McKinney PA, et al. Life-limiting and life-threatening conditions in children and young people in the United Kingdom. Pediatr. 2012;129(4):1-7.

3. Office for National Statistics. Mortality Statistics: deaths registered in 2007. London: Office for National Statistics, 2007.

4. Coad J, Kaur J, Ashley N, et al. Exploring the perceived met and unmet need of life-limited children, young people and families. J Pediatr Nurs. 2015;30(1):45-53.

5. Sidebotham P, Fraser J, Fleming P, et al. Patterns of child death in England and Wales. The Lancet. 2014;384(9946):904-14.

6. Confidential Enquiry into Maternal and Child Health. Why Children Die: a pilot study. London: CEMACH, 2008.

7. Department of Health. Palliative Care Statistics for Children and Young Adults. London: Department of Health; 2007.

8. McCallum DE, Byrne P, Bruera E. How children die in hospital. J Pain Sympt Manage. 2000;20(6):417-23.

9. Ramnarayan P, Craig F, Petros A, et al. Characteristics of deaths occurring in hospitalised children: changing trends. J Med Ethics. 2007;33(5):255-60.

10. Epstein D, Brill JE. A history of pediatric critical care medicine. Pediatr Res. 2005;2005(58):987-96.

11. Widger K, Seow H, Rapoport A, et al. Children's End-of-Life Health Care Use and Cost. Pediatrics. 2017.

12. Plunkett A, Parslow RC. Is it taking longer to die in paediatric intensive care in England and Wales? Arch Dis Child. 2016;101:798-802.

13. World Health Organisation. http://www.who.int/cancer/palliative/definition/en/.

14. Together for Short Lives. Children's Palliative Care Definitions. http://www.togetherforshortlives.org.uk/assets/0000/4089/CPC_definitions.pdf.

15. MacNamara-Goodger K, Feudtner C. History and epidemiology. In: Goldman A, Hain R, Liben S (Ed). Oxford Textbook of Palliative Care for Children, 2nd Ed. Oxford: Oxford University Press; 2012.

16. Hain R, Heckford E, McCulloch R. Paediatric palliative medicine in the UK: past, present, future. Arch Dis Child. 2012;97(4):381-4.

17. Mitchell S, Morris A, Bennett K, Sajid L, Dale J. Specialist paediatric palliative care services: what are the benefits? Arch Dis Child. Published Online First: 04 April 2017. doi: 10.1136/archdischild-2016-3120262017.

18. Rowse V. Home-based palliative care for children: the case for funding. Paediatr Nurs. 2006;18(7):20-4.

19. National Institute for Health and Care Excellence (NICE). End of Life Care for Infants, Children and Young People. https://www.nice.org.uk/guidance/ng61

20. Villanueva G, Murphy MS, Vickers D, et al. End of life care for infants, children and young people with life limiting conditions: summary of NICE guidance. Br Med J. 2016;355.

21. Scottish Government. Strategic Framework for Action on Palliative and End of Life Care. 2015. http://www.gov.scot/Topics/Health/Quality-Improvement-Performance/peolc/SFA

22. Welsh Government. End of life care delivery plan. http://gov.wales/topics/health/nhswales/plans/end-of-life-care/?lang=en: 2014.

23. Department of Health, Social Services and Public Safety, Northern Ireland. Living Matters, Dying Matters: A palliative and end of life care strategy for adults in Northern Ireland www.dhsspsni.giv.uk/publications/living-matters-dying-matters-strategy-2010.

24. Hain RD. Palliative care in children in Wales: A study of provision and need. Palliat Med. 2005;19(2):137-42.

25. Hain RW. Palliative care in children: Who needs it, and why? Acta Paediatrica. 2011;100:4.

26. Baker JN, Levine DR, Hinds PS, et al. Research Priorities in Pediatric Palliative Care. J Pediatr. 2015;167(2):467-70 e3.

27. Mason J. Qualitative Researching. London: SAGE Publishing; 2002.

28. Griffiths F. Research Methods in Healthcare Practice. London: SAGE Publishing; 2009.

29. Ormston R, Spencer L, Barnard M, et al. The Foundations of Qualitative Research. In: Ritchie J, Lewis J, McNaughton Nicholls C, Ormston R, Ed. Qualitative Research Practice. London: SAGE; 2013.

30. Akard T, Gilmer MJ, Friedman DL, et al. From Qualitative Work to Intervention Development in Pediatric Oncology Palliative Care Research. J Pediatr Oncol Nurs. 2013;30(3):153-60.

31. Kendall M, Harris F, Boyd K, et al. Key challenges and ways forward in researching the "good death": qualitative in-depth interview and focus group study. Br Med J. 2007;334(7592):521.

32. Murray SA, Kendall M, Carduff E, et al. Use of serial qualitative interviews to understand patients' evolving experiences and needs. Br Med J. 2009;339.

33. Office for National Statistics. 2011 Census Glossary of Terms Office for National Statistics. London: Office for National Statistics, 2014.

For peer review only

34. Carter B, Coad J, Goodenough T. Community Children's Nursing in England: An appreciative review of CCNs. Department of Health Report. NHS at Home: Community Children's Nursing Services. http://www.dh.gov.uk/en/Publicationsandstatistics/Publications/PublicationsPolicyAndGuidance/DH_1248-98.: 2009.

35. Greig A, Taylor J, MacKay T. Doing Research with Children. A practical guide. London: SAGE; 2008. p. 113-20.

36. Coad J, Gibson F, Horstman M, et al. Be my guest! Challenges and practical solutions of undertaking interviews with children in the home setting. J Child Health Care. 2015;19(4):432-43.

37. Gill P, Stewart K, Treasure E, et al. Methods of data collection in qualitative research: interviews and focus groups. Br Dent J. 2008;204(6):291-5.

38. Lieblich A, Tuval-Mashiach R, Zilber T. Narrative research: reading, analysis and interpretation. London: SAGE, 1998.

39. Bingley A, Thomas C, Brown J, et al. Developing narrative research in supportive and palliative care: the focus on illness narratives. Palliat Med. 2008;22(5):653-8.

40. Burnard P, Gill P, Stewart K, et al. Analysing and presenting qualitative data. Br Dent J. 2008;204(8):429-32.

41. Burnard P. A method of analysing interview transcripts in qualitative research. Nurs Ed Today. 1991;11:461 - 6.

42. Staniszewska S, Boardman F, Gunn L, et al. The Warwick Patient Experiences Framework: patient-based evidence in clinical guidelines. Int J Qual Health Care. 2014;26(2):151-7.

43. Medical Research Council. MRC Ethics Guide: Medical research involving children. London. www.mrc.ac.uk: Medical Research Council, 2004.

44. Shaw C., Brady L-M., Davey C. Guidelines for Research with Children and Young People. London: National Children's Bureau, 2011.

45. Calman L, Brunton L, Molassiotis A. Developing longitudinal qualitative designs: lessons learned and recommendations for health services research. BMC Med Res Method. 2013;13(1):1-10.

46. Hui D, De La Cruz M, Mori M, et al. Concepts and definitions for "supportive care," "best supportive care," "palliative care," and "hospice care" in the published literature, dictionaries, and textbooks. Support Care in Canc. 2013;21(3):659-85.

47. Pastrana T, Ostgarthe C, Elsner F, et al. A matter of definition - key elements identified in a discourse analysis of definitions of palliative care. Palliat Med. 2008;22:222-32.

48. Twamley K, Craig F, Kelly P, et al. Underlying barriers to referral to paediatric palliative care services: knowledge and attitudes of health care professionals in a paediatric tertiary care centre in the United Kingdom. J Child Health Care. 2014;18(1):19-30.

49. Monterosso L, Kristjanson L. Supportive and palliative care needs of families of children who die from cancer: an Australian study. Palliat Med. 2008;22(1):59-69.

50. Morstad Boldt A, Yusuf F, Himelstein BP. Perceptions of the term palliative care. J Palliat Med. 2006;9(5):1128-36.

51. Crocker JC, Beecham E, Kelly P, et al. Inviting parents to take part in paediatric palliative care research: A mixed-methods examination of selection bias. Palliat Med. 2015;29(3):231-40.

52. Hunt A, Coad, J, West, E, et al. The Big Study for Life-limited Children and their Families: Final research report. Bristol: Together for Short Lives, 2013.

For peer review only

For peer review only

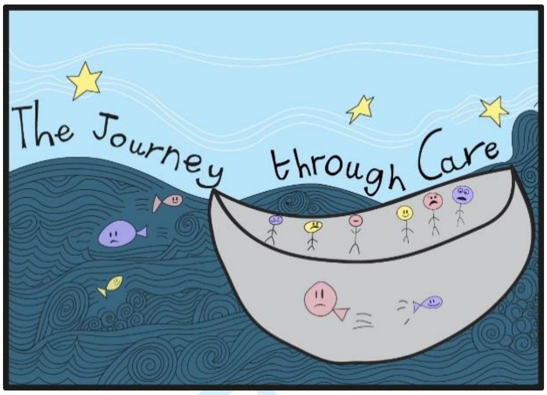

The Journey through Care: Study Information for Families

This information sheet is about a research project which is being completed by Dr Sarah Mitchell at the University of Warwick. The project has been designed to investigate how the NHS provides care to children and young people who are living with serious conditions which may or may not get better; conditions that could be considered life-limiting or life-threatening. The aim of the project is to consider how healthcare services can be improved in the future.

Please read this information sheet to help you decide whether you and / or your child would like to take part. The information sheet will tell you what taking part would mean for you.

What is the project about?

The aim of the project is to understand what children, young people and their families think about the healthcare services that they receive. The project will involve interviews with children and young people and family members who live in the same household. The aim is to consider what works, what doesn't work, and how NHS services can meet the growing needs of seriously ill children, young people and their families in the future.

Why have I been asked to take part?

Children and young people and their families are increasingly sharing their personal stories about illness and their experiences of healthcare services. One example of this are the WellChild Family Bloggers (https://www.wellchild.org.uk/families-area/connect-with-others/). Maybe you have a story too. This research project recognizes that your ideas and experiences, and those of your child, are important. The project is particularly about whether NHS services work, or not, and which services are the most valuable to you.

What is the aim of the project?

The project will collect information (stories) about how services are working now. The stories will be carefully analysed and used to help to improve services for children and young people and their families in the future. A new model of care will be proposed using the research findings which can be used by managers and policy makers. The research will also be written up as academic papers and will be presented at conferences, and local, regional and national service and policy development meetings.

The Journey through Care
IRAS ID: 196816
Leaflet Version: 0.2 1.7.16

For peer review only

What will happen if my child or I decide to take part?

If you could tell your story and would like to take part in the project, please send the attached contact details form back to me. I will then contact you and arrange a time for an interview. I will answer any other questions you have about the study, ask you to sign a consent or agreement form, and arrange an interview with you and your child if that's possible. If you can support your child to take part, there will be a separate form to sign.

Interviews will be arranged at a place and time that is convenient for you. It is anticipated that an interview will last anywhere from 20 – 40 minutes, but there will be no time limit.

You can take part in one, two or three interviews over time if you would like to. This will allow you to gradually tell your story in more detail.

What will happen to the information that is collected?

The interview will, with your consent, be recorded, using a small digital audio recorder. The recordings will be typed up and used later for data analysis by the research team. Your identity will be kept confidential. Any personal information provided or information that identifies other people will be anonymised. Anonymised quotes may be used in publications, for future research and in the development of future educational resources.

The information you give will be only be seen by the researchers. With interviews about healthcare experiences there are some exceptional circumstances that need to be considered, as follows:

1. If there are things that are very difficult to discuss and you need extra support, I will ensure immediate access to this support after the interview, by contacting your clinical team.
2. If any concerns are raised about the standard of care you have received, this will be referred to the clinical team. For your information, Birmingham Children's Hospital Patient Advice and Liaison Service (PALS) can be contacted on 0121 333 8403 or email: pals@bch.nhs.uk. Birmingham Community Healthcare Foundation Trust Patient Experience Team can be contacted on 0800 917 2855 or email: contact.bchc@nhs.net.
3. Although not anticipated, if any very serious concerns arise, for example about child protection, the researcher is bound by the professional code of conduct to report this information to the Clinical Director of the Trust.

Can I have a copy of the recording?

You are welcome to have a copy of the typed out version of the recording of your interviews.

The Journey through Care
IRAS ID: 196816
Leaflet Version: 0.2 1.7.16

For peer review only - http://bmjopen.bmj.com/site/about/guidelines.xhtml

Do I / we have to take part?

No. Participation in this research is voluntary. If you agree to take part, you can change your mind at any time and you do not have to give a reason. If you wish to withdraw consent following the interview, all data relating to the interview, including recordings and transcripts will be destroyed and not included in the study. Your decision about whether to take part or not will in no way influence the care that you receive.

Who can I contact for more details?

If you would like to know more about this project, to take part or if you have any questions please contact:

Sarah Mitchell (Clinical Research Fellow)
University of Warwick Medical School, Gibbet Hill Road, Coventry, CV4 7AL
Email: Sarah.j.mitchell@warwick.ac.uk Tel: 07950202240

You can contact me by email, phone or text and I will get back to you as soon as possible.

What if there is a problem?

If a problem arises or you wish to make a complaint about the conduct of this study or any people involved in it, you may do so by writing to or speaking to the researcher, Sarah Mitchell, who will do her best to answer your queries. If you would prefer not to raise your concern with Sarah, please contact Prof Jeremy Dale (email: Jeremy.Dale@warwick.ac.uk, tel: 02476522891).

The project is funded by a National Institute for Health Research (NIHR) Doctoral Research Fellowship.

Thank-you to the young people's and parents groups from Acorns Children's Hospice and Birmingham Children's Hospital who helped to design this leaflet.

Birmingham Community Healthcare NHS Trust | WARWICK THE UNIVERSITY OF WARWICK | Birmingham Children's Hospital NHS Foundation Trust

The Journey through Care
IRAS ID: 196816
Leaflet Version: 0.2 1.7.16

BMJ Editors Checklist for Qualitative Research

Checklist item	Page No
Was the research question clearly defined?	5
Overall, did the researcher make explicit in the account the theoretical framework and methods used at every stage or the research?	5,11
Was the context clearly described?	3,4,5
Was the sampling strategy clearly described and justified? Was the sampling strategy theoretically comprehensive to ensure the generalisability of the conceptual analysis (diverse range of individuals and settings, for example)?	6
How was the fieldwork undertaken? Was it described in detail?	7-10
Could the evidence (fieldwork notes, interview transcripts, recordings, documentary analysis, etc) could be inspected independently by others: if relevant, could the process of transcription be independently inspected?	15, 16
Were the procedures for data analysis clearly described and theoretically justified? Did they relate to the original research questions? How were themes and concepts identified from the data?	11
Was the analysis repeated by more than one researcher to ensure reliability?	11
Did the investigator make use of quantitative evidence to test qualitative conclusions where appropriate?	N/A
Did the investigator give evidence of seeking out observations that might have contradicted or modified the analysis?	7,8,12,13,14
Was sufficient of the original evidence presented systematically in the written account to satisfy the sceptical reader of the relation between the interpretation and the evidence (for example, were quotations numbered and sources given)?	N/A

For peer review only - http://bmjopen.bmj.com/site/about/guidelines.xhtml

Consolidated criteria for reporting qualitative studies (COREQ): 32-item checklist

Developed from:
Tong A, Sainsbury P, Craig J. Consolidated criteria for reporting qualitative research (COREQ): a 32-item checklist for interviews and focus groups. *International Journal for Quality in Health Care*. 2007. Volume 19, Number 6: pp. 349 – 357

YOU MUST PROVIDE A RESPONSE FOR ALL ITEMS. ENTER N/A IF NOT APPLICABLE

No. Item	Guide questions/description	Reported on Page #
**Domain 1: Research team and reflexivity**		
Personal Characteristics		
1. Inter viewer/facilitator	Which author/s conducted the inter view or focus group?	1
2. Credentials	What were the researcher's credentials? E.g. PhD, MD	1
3. Occupation	What was their occupation at the time of the study?	1
4. Gender	Was the researcher male or female?	1
5. Experience and training	What experience or training did the researcher have?	8
Relationship with participants		
6. Relationship established	Was a relationship established prior to study commencement?	7
7. Participant knowledge of the interviewer	What did the participants know about the researcher? e.g. personal goals, reasons for doing the research	7
8. Interviewer characteristics	What characteristics were reported about the inter viewer/facilitator? e.g. Bias, assumptions, reasons and interests in the research topic	13-14
**Domain 2: study design**		
Theoretical framework		
9. Methodological orientation and Theory	What methodological orientation was stated to underpin the study? e.g. grounded theory, discourse analysis, ethnography, phenomenology, content analysis	6-7
Participant selection		
10. Sampling	How were participants selected? e.g. purposive, convenience, consecutive, snowball	7
11. Method of approach	How were participants approached? e.g. face-to-face, telephone, mail, email	7
12. Sample size	How many participants were in the study?	11

For peer review only

13. Non-participation	How many people refused to participate or dropped out? Reasons?	N/A
Setting		
14. Setting of data collection	Where was the data collected? e.g. home, clinic, workplace	8-9
15. Presence of non-participants	Was anyone else present besides the participants and researchers?	9
16. Description of sample	What are the important characteristics of the sample? e.g. demographic data, date	7
Data collection		
17. Interview guide	Were questions, prompts, guides provided by the authors? Was it pilot tested?	9-10
18. Repeat interviews	Were repeat inter views carried out? If yes, how many?	11
19. Audio/visual recording	Did the research use audio or visual recording to collect the data?	11
20. Field notes	Were field notes made during and/or after the inter view or focus group?	11
21. Duration	What was the duration of the inter views or focus group?	N/A
22. Data saturation	Was data saturation discussed?	11
23. Transcripts returned	Were transcripts returned to participants for comment and/or correction?	12
**Domain 3: analysis and findings**		
Data analysis		
24. Number of data coders	How many data coders coded the data?	12
25. Description of the coding tree	Did authors provide a description of the coding tree?	12
26. Derivation of themes	Were themes identified in advance or derived from the data?	12
27. Software	What software, if applicable, was used to manage the data?	12
28. Participant checking	Did participants provide feedback on the findings?	N/A
Reporting		
29. Quotations presented	Were participant quotations presented to illustrate the themes/findings? Was each quotation identified? e.g. participant number	N/A
30. Data and findings consistent	Was there consistency between the data presented and the findings?	N/A
31. Clarity of major themes	Were major themes clearly presented in the findings?	N/A
32. Clarity of minor themes	Is there a description of diverse cases or discussion of minor themes?	N/A

Once you have completed this checklist, please save a copy and upload it as part of your submission. When requested to do so as part of the upload process, please select the file type: *Checklist*. You will NOT be able to proceed with

For peer review only

submission unless the checklist has been uploaded. Please DO NOT include this checklist as part of the main manuscript document. It must be uploaded as a separate file.

For peer review only

BMJ Open

The Journey through Care: Study protocol for a longitudinal qualitative interview study to investigate the healthcare experiences and preferences of children and young people with life-limiting and life-threatening conditions, and their families in the West Midlands, UK.

For peer review only

Journal:	*BMJ Open*
Manuscript ID	bmjopen-2017-018266.R1
Article Type:	Protocol
Date Submitted by the Author:	15-Aug-2017
Complete List of Authors:	Mitchell, Sarah; Warwick Medical School<br>Slowther, Anne-Marie; University of Warwick, Warwick Medical School<br>Coad, Jane; Coventry University<br>Dale, Jeremy; University of Warwick, Warwick Medical School
**Primary Subject Heading**:	Palliative care
Secondary Subject Heading:	Health services research, Paediatrics, Patient-centred medicine, Qualitative research
Keywords:	Organisation of health services < HEALTH SERVICES ADMINISTRATION & MANAGEMENT, Paediatric palliative care < PAEDIATRICS, PALLIATIVE CARE, QUALITATIVE RESEARCH

SCHOLARONE™
Manuscripts

The Journey through Care: Study protocol for a longitudinal qualitative interview study to investigate the healthcare experiences and preferences of children and young people with life-limiting and life-threatening conditions, and their families in the West Midlands, UK.

Mitchell S[1], Slowther A-M[1], Coad J[2], Dale J[1].

Corresponding Author: Sarah Mitchell MB ChB FRCGP MSc

National Institute of Health Research Doctoral Research Fellow

Sarah.j.mitchell@warwick.ac.uk; 07950202240; @MacGPSarah

Author affiliations:

1. Warwick Medical School, University of Warwick, Gibbet Hill Road, Coventry, CV4 7AL
2. Faculty of Health and Life Sciences, Coventry University, Priory Street, Coventry, CV1 5FB.

Word counts

- Abstract: 460
- Protocol: 3994

Tables: 5

Boxes: 1

Colour Figures: 2

For peer review only

Abstract

INTRODUCTION

The numbers of children and young people living with life-limiting and life-threatening conditions is rising. Providing high quality, responsive healthcare for them and for their families presents a significant challenge. Their conditions are often complex and highly unpredictable. Palliative care is advocated for people with life-limiting and life-threatening conditions, but these services for children are highly variable in terms of availability and scope. Little is known about the lived experiences and preferences of children and their families in terms of the palliative care that they do, or do not, receive.

This study aims to produce an in-depth insight into the experiences and preferences of such children and families in order to develop recommendations for the future provision of services. The study will be carried out in the West Midlands, UK.

METHODS AND ANALYSIS

A qualitative study comprising longitudinal interviews over a ??12 month period with children (aged 5-18 years) living with life-limiting or life-threatening conditions, their family members.

Data analysis will start with thematic analysis, followed by narrative and cross-case analysis to examine changing experiences and preferences over time, at the family level and within the wider healthcare system.

Patient and Public Involvement (PPI) has informed the design and conduct of the study. Findings will be used to develop recommendations for an integrated model of palliative care for children in partnership with the PPI group.

ETHICS AND DISSEMINATION

Ethical approval was granted in September 2016 by the NHS Health Research Authority (IRAS ID: 196816, REC reference: 16/WM/0272).

Findings will be of immediate relevance to health care providers, policy makers, commissioners and voluntary sector organisations in the UK and internationally. Reports will be prepared for these audiences, as well as for children and their families, alongside academic outputs.

STRENGTHS AND LIMITATIONS

- An in-depth, contextual, longitudinal qualitative study with multiple child and family member stories captured over time.

For peer review only

- New insights will be provided because all of the children and families included in the study could benefit from palliative care as it is currently defined, however not all will have had conversations about this or have been referred to specialist palliative care services. Findings will focus on healthcare, but there is wider applicability and relevance to social care and joint planning of services.
- A diverse study population in terms of age, clinical condition, cultural background and family structure will allow detailed consideration of the role of healthcare services in effectively recognising and supporting children and families with their individual needs. However all will speak English.
- Neonates, pre-school children and young people at transition (over the age of 18 years) are all excluded and warrant research in their own right.
- There are multiple potential sources of bias which will be addressed throughout the study, including recruitment bias and the unconscious bias of the researcher.

Author's contributions

SM drafted the protocol with regular academic supervision from JD, A-M S and JC. The study was conceptualised by SM, JD, A-MS and JC, informed and guided by Patient and Public Involvement. The protocol incorporates peer review feedback received during the application process for the NIHR Doctoral Research Fellowship, and the MPhil to PhD upgrade panel at the University of Warwick. JD, A-MS and JC have all reviewed the protocol for intellectual content. All authors have reviewed and agreed this version.

Funding statement

This work is supported by a National Institute of Health Research Doctoral Research Fellowship (Dr Sarah Mitchell DRF-2014-07-065)

Competing interests

The authors have no competing interests.

For peer review only

Introduction

Children and young people with life-limiting conditions and life-threatening conditions represent a growing concern in healthcare (1). With advances in clinical practice, the numbers of children living with these conditions is rising (1-3). The nature of their conditions is complex and unpredictable; the risk of a sudden deterioration and death is an everyday reality for many. Family carers can experience enormous emotional, physical and financial pressures (4).

Research suggests that families of children with life-limiting and life-threatening conditions wish for continuous, holistic healthcare, with the option that this is delivered in the home environment (4). However most children who die have a pre-existing life-limiting condition (5), and most die in hospital (6, 7), most frequently in an intensive care environment, where the mode of death is often withdrawal or limitation of life-sustaining treatments (8-10). The length of stay in the intensive care unit before death is increasing , and the costs of hospital care at the end of life are significant (11, 12). This situation presents complex clinical and ethical dilemmas at individual, organizational and societal levels.

Palliative care is an approach to care that is advocated for all people who live with a life-limiting or life-threatening condition. Current definitions of palliative care are broad (box 1), which can cause difficulties and lack of clarity for those designing and commissioning specific services.

Box 1: Children's Palliative Care Definitions (13, 14):

Palliative care for children with life-threatening conditions is defined by the World Health Organisation as "a special, albeit closely related field to adult palliative care; the principles apply to other paediatric chronic disorders:

- The active total care of the child's body, mind and spirit, and support to the family.
- It begins when illness is diagnosed, and continues regardless of whether or not a child receives treatment directed at the disease.
- Health providers must evaluate and alleviate a child's physical, psychological, and social distress.
- Effective palliative care requires a broad multidisciplinary approach that includes the family and makes use of available community resources; it can be successfully implemented even if resources are limited.
- It can be provided in tertiary care facilities, in community health centres and even in children's homes"

The UK national charity for paediatric palliative care, Together for Short Lives, defines palliative care for children with life-limiting conditions as "an active and total approach to care, from the point of diagnosis or recognition, embracing physical, emotional, social and spiritual elements through to death and beyond. It focuses on enhancement of quality of life for the child and support for the family and includes the management of distressing symptoms, provision of short breaks and care through death and bereavement".

For clarity, in this paper children and young people will be referred to throughout as “children”.

WHICH CHILDREN?

There has been a range of previous work concerned with the identification of clinical conditions where palliative care could be beneficial (15, 16), and the following categorisation is provided by Together for Short Lives (14):

- *Group 1:* life-threatening conditions where access to palliative care services is necessary alongside attempts at curative treatment and / or if treatment fails, such as cancer.
- *Group 2:* conditions such as Duchenne muscular dystrophy, where premature death is inevitable, but where there may be long periods where the child is well.
- *Group 3:* progressive conditions without curative treatment options, such as Batten disease.
- *Group 4:* irreversible but non-progressive conditions, with complex disabilities and healthcare needs which lead to increased likelihood of premature death, such as severe brain injury.

ORGANISATIONAL ISSUES

Currently there is wide geographic variation in terms of paediatric palliative care services, and a poorly understood range of commissioning arrangements to support these services. Many services exist as a result of significant contributions from the voluntary sector (including children’s hospices), through the efforts of motivated individuals, and through non-recurring funding opportunities rather than the implementation of national policy (17). A significant development is the emergence of paediatric palliative medicine as a sub-speciality of paediatrics (18, 19).

Effective palliative care services for children require strong partnerships between providers, and may require cross-boundary, collaborative commissioning between the statutory and voluntary sectors (20). In the UK, palliative care for CYP has specifically been included in national policy , a service specification for paediatric palliative care exists and NICE (National Institute for Health and Care Excellence) Guidelines for End-of-Life Care for Infants, Children and Young People were published in 2016 (21-25).

RATIONALE FOR RESEARCH

Despite the range of recommendations for the provision and development of paediatric palliative care services, there remains a lack of research evidence to support the implementation of these guidelines (1, 19, 26-28). The proposed study is designed to address this gap by exploring the strengths and limitations of current healthcare service provision and the expectations and experiences of those who could potentially benefit from this approach to care.

For peer review only - http://bmjopen.bmj.com/site/about/guidelines.xhtml

RESEARCH QUESTIONS

1. How do children with life-limiting and life-threatening conditions and their family members perceive healthcare services, and in particular "palliative care"?
2. What are the experiences and preferences of children living with a life-limiting or life-threatening condition, and/or their families, in relation to the delivery of healthcare services?
3. What are the facilitators and barriers to the delivery of palliative care for children, and how might these be overcome?
4. What should an integrated model of palliative care for children look like?

ONTOLOGY AND EPISTEMOLOGY

The study is embedded in the relativist paradigm (29) and draws upon the philosophical models of realism and constructionism, which dictate that reality exists and is actively "constructed" by human beings through actions in a social context, rather than being passively received by them (29, 30). The research aims to provide an in-depth insight into the complex reality that is the experience of healthcare from the perspective of children with life-limiting and life-threatening conditions and their families, and to examine how this relates to current policy and service delivery, a "causal" intellectual puzzle (Figure 1) (31).

Figure 1: Healthcare policy, service design and delivery and their influence on the child and family experience as a causal intellectual puzzle.

METHODOLOGY AND THEORETICAL PERSPECTIVE

The theoretical perspectives which will guide the study are expected to be modified, reviewed and added to during the course of the research (32), but will include critical realism, an emerging approach in healthcare research including health sector management (33-35), and one that has been related closely to general practice (36). The researcher (SM) is a general practitioner (GP) with previous qualitative research experience.

In order to conduct an exploration of the experiences of healthcare from the perspective of children with life-limiting and life-threatening conditions and their families, we will adopt qualitative research methods and a narrative-based approach, suitable for complex, emotional subject areas (37, 38). Active listening, reflection, a flexible approach and insight into the narratives being co-constructed between participant and researcher will be necessary throughout.

The research plan has been informed by review of relevant literature, patient and public involvement (PPI) work and advice from local experts via the West Midlands Paediatric Palliative Care Network.

For peer review only - http://bmjopen.bmj.com/site/about/guidelines.xhtml

Methods and Analysis

This is the protocol for an in-depth longitudinal qualitative study using semi-structured interviews with school-aged children (5-18 years) and one or two of their household family members.

Benefits of longitudinal studies include being able to describe the changing needs of the children and their families, and their experience of services, over time (39), and enabling rapport to build between researcher and participant.

Neonates, pre-school children and young people aged over the age of 18 years are excluded from this study. Specific issues around healthcare services arise when considering neonatal care and young people who are making the transition from paediatric to adult services, both of which warrant research in their own right. Research methods would need to be tailored to interview pre-school children; this is also an area for potential future research.

SAMPLING AND RECRUITMENT

Recruitment to a study of this nature depends on many factors, including the clinical condition of the child, conflicting demands on the family's time, and the motivation and understanding of their clinical teams. Recruitment began following ethical approval in October 2016 and will continue until January 2018.

The approach to participants is through:

1. direct invitation via their clinical team
2. leaflets and posters displayed in public areas in the hospital (such as notice boards on wards and in outpatients).

The research will be introduced to clinical teams in both the hospital and the community through formal presentations at departmental meetings and to individual clinicians at their request, as well as to the paediatric palliative care network. The researcher, SM, will undertake a period of shadowing with clinical teams, on hospital wards, in outpatient clinics and in the community.

Potential participants will be provided with a participant information sheet, with details of the researcher, the project, how to get involved, and a contact telephone number and email address.

Inclusion and exclusion criteria are outlined in Table 1. Our aim initially is to purposively sample children so that each of the four Together for Short Lives categories are represented. However, since children live with such individual and highly complex conditions we anticipate that achieving this may be difficult. The study population will therefore be children with life-limiting or life-threatening conditions, aged from 5-18 years, and their family members, some of whom have experience of a palliative care service, and some who do not.

For peer review only

The study has been carefully designed to ensure that all of the children have the opportunity to participate and that wherever possible the views of the child are included, by tailoring each individual interview to their needs and capabilities (including the consent and agreement process). This may include having a learning disability or communication difficulties associated with their condition.

Ethical approval has been granted for the recruitment of 12-14 families to take part in a series of interviews (longitudinal interviews). The aim is to continue to conduct interviews until data saturation is achieved (40), however given the uniqueness and individuality of the stories of children and families, it is possible that new themes will continue to emerge such that data saturation is impossible. We will aim for saturation of the main themes that emerge from the data, and identify emergent themes, which may form the basis of future research.

Table 1: Inclusion and Exclusion Criteria

Inclusion Criteria	1. Children aged 5-18 yrs (school age) with a life-limiting or life-threatening condition who are under the care of the Community Children's Nursing Team and/or the Children's Hospital and who either:<br>• receive palliative care services<br>• are aware of (have had discussions about) palliative care services<br>• are living with relapsing or refractory disease<br>• or have had a life-threatening episode (admission to the Paediatric Intensive Care Unit (PICU))<br><br>2. Their family members, who live in the same household (41).
Exclusion Criteria	• Children aged < 5 years and > 18 years.<br>• Families of children < 5 years and > 18 years old<br>• Children and families with whom the research team has clinical contact<br>• Children and / or families who do not wish to participate.<br>• Children who are too unwell will not be approached for interview, but their family members may still participate if they wish to. The researcher will take advice from parents about when an individual child is "too unwell" to take part.<br>• Children who are unable to participate in a conversational interview for any reason related to their condition will not be approached for interview, but their family members may participate if they wish to.<br>• Children and families who are unable to provide informed

For peer review only - http://bmjopen.bmj.com/site/about/guidelines.xhtml

	consent in English will not be approached for interview.

INTERVIEW PLAN

Interviews will be carried out by SM, a researcher who is also a GP with advanced communication skills training and previous experience in qualitative interviewing. According to the preference of participants, interviews will be conducted with individuals, or with the child and parent together, in their preferred location. One or two family members will be interviewed in each family, either individually or together, depending on their preference and what is most convenient for them.

Interviews will be open and conversational, using a blended approach of interview techniques, with passive interviewing allowing the participant space and time to tell their story (narrative), and more active techniques, including appreciative inquiry, which asks "what works well?" and "why does it work well?" (4, 42), employed. A topic guide (table 2) will guide the interview; this will continue to develop iteratively throughout the research, with adaptations made during each interview and in response to each individual participant.

Table 2: Topic Guide

For all families	For those aware of "palliative care"
**Introduction**<br><br>**Please tell me your story, in any way that you can / want to**<br><br>**Please tell me the story of you**<br><br>*Your Story*<br><br>**Please can you tell me a about you?**<br><br>**Your family?**<br><br>**Your child(ren)**<br><br>**What is important to you?**<br><br>**What do you like to do?**	*Palliative care and you (if appriopriate)*<br><br>**Do you have "palliative care" services?**<br><br>Have you ever heard the term "palliative care"?<br><br>What does that mean to you?<br><br>What do you receive those services for?<br>What do these services provide for you?<br>Does it matter what a service is called?<br><br>Do you receive services from the hospice?<br><br>**Can you tell me how you came to receive palliative care / know the palliative care nursing team / the hospice?**<br><br>When were you referred?<br><br>Who brought it up / made the referral?

For peer review only

**Which places are important to you?**<br><br>**Where do you spend your time?**<br><br>**Which services are involved in your care?**<br><br>**Who comes to see you?**<br><br>**What do they do?**<br><br>What's helpful?<br><br>What's not?<br><br>**Which healthcare professionals do you consider to be key in the delivery of your care?**<br><br>**What works best?**<br><br>Which services / professionals are most helpful?<br><br>Which services / professionals do you value most?<br><br>**What doesn't work?**<br><br>**How do you think services could be improved?**<br><br>**Do you talk to other children / young people / families about your healthcare / services"?**<br><br>What do you tell your friends?<br><br>What tends to come up in these discussions?<br><br>Would you recommend these services to others?	How was this discussed with you?<br><br>How was that for you / your family?<br><br>**Do you think that medical / nursing staff receive enough training in this area?**<br><br>What makes you think that?<br><br>**Anything else?**

For peer review only

For interviews with children, a range of techniques will be used including de-personalising questions, developing a narrative in the third person, and using props and toys to encourage story-telling. Arts-based activities will be used, where appropriate, as a mutual point of focus for the researcher and participant, or as a focus of the interview, as in the draw-write-tell technique (43). PPI advice has been sought on the format of interviews for children (Table 3).

Table 3: Feedback from PPI groups on interview plans

January 2016	*"Those who are passionate about improving palliative care will take part regardless of how sensitive this may be"*
July 2016	*"Remember young people who are seriously ill are more mature, they have to grow up"*<br>*"Keep it simple as often a child will openly speak anyway"*<br>*"'Do you talk about it to your friends?' is a good question, a good way to talk to most ages."*<br>*"Children are more eloquent, mature and more capable than you think"*
October 2016	*"Use pictures and images, more emojis"*
February 2017	*"Doesn't make me uncomfortable as I think it is very important and relevant"*

Each interview will be audio-recorded, with field notes made to include any additional comments from the child or family made once the audio-recording has stopped (44), reflections on the interview and observation of the family situation, environment, behaviour, and any other interactions that may take place (for example with other members of the family and clinical staff in hospital or on the phone).

Participants will be asked whether they would like to participate in interviews that will take place over a period of up to 12 months. These are intended to allow the identification of common themes over time and for theories generated through analysis of earlier interviews to be tested out during later interviews (39). The time intervals between interviews will be individually agreed, depending on the child and family circumstances. The method of communication with each family will also be individually agreed (phone or email). Up to three interviews with each participant are aimed for.

We anticipate practical challenges with conducting longitudinal interviews relating to fluctuations and changes in the clinical condition of each child. Depending on their condition, some children will respond well to treatments and get better. Others may suffer unexpected complications of their

For peer review only

condition or treatment, and some may suffer deteriorations which bring about the possibility of dying. To manage the research in this context, we will check the family understanding of the situation before every interview. On occasions, interviews may need to be postponed and rearranged at late notice due to a change in circumstances.

For children who are unable to participate in interviews due to their condition, family members will be interviewed. Children and families are under no obligation to take part in follow-up interviews if they do not wish to. In these cases, and with their consent, data from previous interviews will still be included in the study.

DATA ANALYSIS

Interviews will be transcribed verbatim, and NVivo used for data handling. Analysis of interview transcripts and field notes will commence alongside data collection, with an initial broad thematic analysis. All data will be coded, and codes grouped into broad overarching themes.

This initial analysis will be followed by an in-depth, narrative analysis, using structure-form analysis to examine not just what is being said, but how it is being said, and to position narratives in the context of palliative care from a micro (immediate clinical team), meso (local organisation) and macro (wider healthcare system) level perspective (45, 46).

The collection of longitudinal data allows for innovative approaches to be taken in data analysis (39). Matrices will be developed to identify key times for families and identification of cross-cutting themes at these times, for example the time of diagnosis, an admission to intensive care or referral to a palliative care team.

Peer review and respondent validation will take place throughout the data analysis as follows (47, 48):

1. Peer review: SM will code all of the data. A selection of transcripts will be reviewed and independently coded by other members of the research team in order to decrease lone researcher bias (47). The coding frameworks will be discussed and compared, allowing further development of categories and themes.
2. Respondent validation: By returning to participants to conduct longitudinal interviews, there is opportunity to check, validate or refute emergent themes from the initial data analysis.

HEALTHCARE PROFESSIONAL PERSPECTIVES

There are 12 paediatric palliative care networks in the UK, which include professionals from a range of organisations within paediatric palliative care. Several have patient and family representatives. Arrangements will be made to present initial study findings and work in progress to four of the UK

For peer review only - http://bmjopen.bmj.com/site/about/guidelines.xhtml

networks at existing meetings to test out emerging themes and the generalizability of the research findings. Through this process there will be opportunity to compare multiple perspectives which will inform and guide the formulation of recommendations for healthcare services in the future (49).

An expression of interest email will be circulated to network chairs via Together for Short Lives, and arrangements made to attend meetings from networks who respond. Audio-recorded focus group discussions will be carried out at those meetings by SM.

Patient and Public Involvement

PPI has been integral to the design and conduct of the study. Members of existing groups at a children's hospital and children's hospice have provided advice on the study proposal and design. Smaller groups have been recruited for specific activities, including conference presentations. Group members range in age from 12 to 22 years. PPI will continue throughout the project, with the aim of co-producing the recommendations for the model of care. This will involve structured group sessions during which anonymised findings of the data analysis will be presented to the group for feedback and comment. A patient experience framework will be used to structure the discussion and to build recommendations (50).

Table 4: PPI activities

Completed PPI activities	• Developing the original research proposal<br>• Advising on the language used in the study (suggesting a change in the title from "Palliative Care for Children and Young People: What? When? How?" to "The Journey through Care".<br>• Developing participant resources including leaflets for older and younger children<br>• Interview design, including suggesting how questions could be phrased and asked<br>• Providing family perspectives to a literature review, and becoming a co-author on the paper (19).<br>• Taking part in oral presentations at regional conferences
Work in progress	• Designing conference posters and presentations for national conferences<br>• Working as co-researchers to carry out a survey study to investigate understanding of the term "palliative care" for children and young people, and healthcare professionals
Future plans	• Working with a project-specific group to explore the findings of the research study and develop recommendations for a new model of care.

	• Dissemination projects including conference presentations, posters, website design, use of social media, infographics and films.

Strengths and Limitations

The strength of this study lies in the in-depth, contextual qualitative nature of the data, with multiple child and family member stories captured over time. Our anticipated study population is diverse in terms of age, clinical condition, cultural background and family structure, allowing detailed consideration of the role of healthcare services in effectively recognising and supporting children and families with their individual needs. All of the children and families included in the study could benefit from palliative care as it is currently defined (14), however not all will have had conversations about this with their clinicians, or been referred to specialist palliative care services. Given the nature of their clinical conditions, including for some the inability to communicate verbally or deterioration in their health, recruitment and retention within the study is likely to become a challenge and will require a reflexive, flexible approach.

Potential limitations in the study include our exclusion of neonates, pre-school children and young people at transition (over the age of 18 years). These groups all warrant research in their own right. Given the time and resource constraints of the study, all interviews will be carried out in English. Further research into the experiences of CYP and families who cannot communicate in English is necessary. There will be ongoing consideration of sources of bias. Recruitment bias is being addressed by aiming for a diverse sample and providing access to project information independent of the clinical teams. Data saturation will be sought during data analysis, with an ongoing process of reflection and peer review to address any possible unconscious bias of the researcher (SM).

Ethics and Dissemination

Research with children raises ethical and legal considerations around recruitment, consent, and data collection (51, 52). In addition research regarding palliative and end-of-life care can be emotionally demanding and distressing for those involved. There are also particular ethical issues to consider given the longitudinal nature of the study(53).

We are recruiting children and families who are potentially vulnerable and may be experiencing considerable distress. The justification for our approach is that children and their families in this situation are rarely asked about their experiences, but talking to them and understanding their

For peer review only

experiences is essential in order to be able to design and develop services that respond to their actual needs. Here we summarise our approach to the ethical issues the study raises (Figure 2).

Figure 2: Ethical issues in longitudinal qualitative research for children and families in palliative care

LANGUAGE

Published literature suggests that the term "palliative care" is poorly understood and perceived negatively (54-58), a view confirmed by our PPI group. The scope of our study is therefore to investigate the experiences of children with "life-limiting", "life-threatening" and "conditions which may or may not get better", whether or not they have heard of palliative care or receive care from specialist services. "Palliative care" will be avoided in participant information sheets and interviews, unless individuals are already familiar with palliative care services or bring it up themselves.

RECRUITMENT

There is an ethical challenge in terms of potential coercion to the study by clinicians who know the family well. Clinicians will therefore only provide study information but will not actively recruit families; the initial expression of interest is from the family to the researcher. The researcher (SM) will then discuss the study in person or by phone with the child and their family member(s) and answer any questions before arranging a time for interview. Participants will be made aware that they can decline to take part or to withdraw at any stage without having to give a reason. Interviews are only carried out at a time that is mutually agreed and minimises any potential inconvenience or intrusion.

Equity of access to the study

Recruitment through clinical teams is widely used in palliative care research but may be limited by "gatekeeping" (59). There may also be families who wish to participate who do not find out about the study through their clinical team. In order to address this, we have designed posters for display on hospital wards and in outpatients, and at the local children's hospice, and a paragraph for organisational newsletters. These provide the direct contact details for the researcher (email, text or phone).

The study setting is Birmingham, UK, a city where the population is highly diverse in terms of family situation and multiculturalism. Over 50% of families with a child known to palliative care services in Birmingham and Solihull are from black or minority ethnic backgrounds (60). Many of these families speak English as a first or second language , so within the time and resource constraints of this study, interviews will be carried out with those who can provide informed consent and take part in an interview in English.

For peer review only

CONSENT

Consent for the study raises ethical and legal issues with children who are under the age of 16 years and / or do not have the capacity to consent. We will aim for written and / or verbal consent and agreement from every individual for every interview.

For children under the age of 16, written consent will be obtained from the parent and then verbal or written agreement obtained from the child.

In keeping with the Mental Capacity Act, there is an assumption of capacity in young people aged 16 years and over, so they will be asked for consent first, followed by agreement from their parent(s). Parental agreement is not a legal requirement, but conducting an interview with a young person about a potentially difficult subject without the knowledge or agreement of their parents is an ethical concern. If there is a concern that the young person lacks capacity or is considered particularly vulnerable, for example with a learning disability, parents will be asked to provide verbal and written consent in addition to the young person's agreement.

Parental consent is required for any interview to be carried out in the family home (52).

For a child on a full care order, social worker consent would replace that of parental consent. Where possible parental consent / agreement will also be sought.

INTERVIEWS

Subject areas discussed during interviews may cause distress to participants, and recruitment may occur soon after sensitive conversations. We have designed the study to ensure that the risks and burden associated with taking part in the study are minimal.

Qualitative interviews will be informal and reflexive to the needs of the participant. In the event that a participant experiences any difficulties during the interview, such as tiredness or distress, the interview will be halted, and if necessary brought to an end. Adequate time will be given for debrief, and the researcher will provide information about local resources for support if necessary. Interviews will be carried out at a time and in a location that is convenient to the individual participant. If this is in hospital, the researcher (SM) will liaise closely with clinical teams so that the research does not interfere with routine clinical care and ward work.

Longitudinal interviews

Family views and understanding of what might happen next as a result of the condition of the child will be discussed sensitively, and any follow-up interviews scheduled around possible further treatments. If it seems likely that there will be a deterioration in the condition of the child, this is

For peer review only - http://bmjopen.bmj.com/site/about/guidelines.xhtml

explored carefully and an agreement made with the individual family about whether they want to continue to participate in the study.

ANONYMITY AND CONFIDENTIALITY

All interview data will be anonymised with personal identifiers removed. Any qualitative interview data that could identify child, families or any professionals involved in their care because of the individuality and context of the narrative is included in the data analysis, but will be excluded from reporting.

Field notes and anonymised interview transcripts will be stored securely on a password protected university hard drive.

MINIMISING HARM TO THE RESEARCHER

There is a need for clearly defined boundaries for a researcher – participant relationship in a longitudinal study of this type. It will be made clear to participants at the time of consent that it is not the role of the researcher to provide personal support or clinical advice. With the risk of emotional distress for the researcher, plans to ensure adequate support through regular academic supervision and access to a counsellor are in place.

SERIOUS CONCERNS AND SAFEGUARDING

If information contained in a participant's response indicates a serious clinical or safeguarding concern or an issue which may jeopardise the safety of the participant or another person, this will be escalated appropriately in line with the protocols of the community or hospital trusts. This may on very rare occasions necessitate a breach of participant confidentiality in order to maintain their safety. Participants will be informed of any disclosure and to whom it is made.

ETHICAL APPROVAL

Ethical approval was granted by the UK Health Research Authority on 14th September 2016 (IRAS ID: 196816, REC reference: 16/WM/0272, Sponsor: University of Warwick).

DISSEMINATION PLAN

The research is embedded in plans for impact. Table 5 outlines our planned outputs. We will work on traditional academic and clinical outputs, including manuscripts with the results of the study for publication in a peer-reviewed journal. Simultaneously work will be carried out with the PPI group to plan innovative, accessible outputs for patients, the public and commissioners which will include infographics and film based reports outlining our recommendations.

Table 5: Planned outputs from the research

For peer review only

Academic / Clinical audiences	Patient, public and policy-maker audiences
• PhD Thesis<br>• Peer reviewed publications<br>• Presentations at national and international conferences	• A report prepared for participants and PPI volunteers<br>• The development of guidance for commissioners and providers<br>• The development of resources that are accessible to patients and families

References

1. Fraser LK, Miller M, Hain R, et al. Rising national prevalence of life-limiting conditions in children in England. Pediatrics. 2012;129(4):e923-9.
2. Fraser LK, Parslow RC, McKinney PA, et al. Life-limiting and life-threatening conditions in children and young people in the United Kingdom. Pediatr. 2012;129(4):1-7.
3. Office for National Statistics (2007). Mortality Statistics: deaths registered in 2007.
4. Coad J, Kaur J, Ashley N, et al. Exploring the perceived met and unmet need of life-limited children, young people and families. J Pediatr Nurs. 2015;30(1):45-53.
5. Sidebotham P, Fraser J, Fleming P, et al. Patterns of child death in England and Wales. The Lancet. 2014;384(9946):904-14.
6. Confidential Enquiry into Maternal and Child Health (2008). Why Children Die: a pilot study. London: CEMACH, 2008.
7. Department of Health (2007). Palliative Care Statistics for Children and Young Adults. London: Department of Health; 2007.
8. McCallum DE, Byrne P, Bruera E. How children die in hospital. J Pain Sympt Manage. 2000;20(6):417-23.
9. Ramnarayan P, Craig F, Petros A, et al. Characteristics of deaths occurring in hospitalised children: changing trends. J Med Ethics. 2007;33(5):255-60.
10. Epstein D, Brill JE. A history of pediatric critical care medicine. Pediatr Res. 2005;2005(58):987-96.
11. Widger K, Seow H, Rapoport A, Chalifoux M, Tanuseputro P. Children's End-of-Life Health Care Use and Cost. Pediatr. 2017.
12. Plunkett A, Parslow RC. Is it taking longer to die in paediatric intensive care in England and Wales? Arch Dis Child. 2016;101:798–802.
13. World Health Organisation. http://www.who.int/cancer/palliative/definition/en/.
14. Together for Short Lives. Children's Palliative Care Definitions. http://www.togetherforshortlives.org.uk/assets/0000/4089/CPC_definitions.pdf.

For peer review only

15. Hain R, Devins M, Hastings R, et al. Paediatric palliative care: development and pilot study of a 'Directory' of life-limiting conditions. BMC Palliat Care. 2013;12(1):43.
16. Noyes J, Edwards RT, Hastings RP, et al. Evidence-based planning and costing palliative care services for children: novel multi-method epidemiological and economic exemplar. BMC Palliat Care. 2013;12(1):18.
17. MacNamara-Goodger K, Feudtner C. History and epidemiology. In: Goldman A, Hain R, Liben S, Ed. Oxford Textbook of Palliative Care for Children 2nd ed. Oxford: Oxford University Press; 2012.
18. Hain R, Heckford E, McCulloch R. Paediatric palliative medicine in the UK: past, present, future. Arch Dis Child. 2012;97(4):381-4.
19. Mitchell S, Morris A, Bennett K, et al. Specialist paediatric palliative care services: what are the benefits? Arch Dis Child. doi: 10.1136/archdischild-2016-3120262017.
20. Rowse V. Home-based palliative care for children: the case for funding. Paediatric nursing. 2006;18(7):20-4.
21. National Institute for Health and Care Excellence (NICE). End of Life Care for Infants, Children and Young People. https://www.nice.org.uk/guidance/ng61
22. Villanueva G, Murphy MS, Vickers D, et al. End of life care for infants, children and young people with life limiting conditions: summary of NICE guidance. Br Med J. 2016;355.
23. Scottish Government. Strategic Framework for Action on Palliative and End of Life Care. 2015. http://www.gov.scot/Topics/Health/Quality-Improvement-Performance/peolc/SFA
24. Welsh Government. End of life care delivery plan. http://gov.wales/topics/health/nhswales/plans/end-of-life-care/?lang=en: 2014.
25. Department of Health, Social Services and Public Safety, Northern Ireland. Living Matters, Dying Matters: A palliative and end of life care strategy for adults in Northern Ireland www.dhsspsni.giv.uk/publications/living-matters-dying-matters-strategy-2010.
26. Hain RW. Palliative care in children in Wales: A study of provision and need. Palliat Med 2005;19(2):137-42.
27. Hain RW. Palliative care in children: Who needs it, and why ? Acta Paediatrica 2011;100:4.
28. Baker JN, Levine DR, Hinds PS, et al. Research Priorities in Pediatric Palliative Care. J Pediatr. 2015;167(2):467-70 e3.
29. Crotty M. The Foundations of Social Research: Meaning and perspective in the research process. Sydney: Allen & Unwin; 1998.
30. Ormston R, Spencer L, Barnard M, et al. The Foundations of Qualitative Research. In: Ritchie J, Lewis J, McNaughton Nicholls C, Ormston R, Ed. Qualitative Research Practice. London: SAGE; 2013.
31. Mason J. Qualitative Researching. London: SAGE Publishing; 2002.
32. Ragin C, Becker H. What is a case? Exploring the foundations of social inquiry: Cambridge University Press; 1992.
33. Schiller CJ. Critical realism in nursing: an emerging approach. Nurs Philos. 2016;17(2):88-102.
34. Walsh D, Evans K. Critical realism: an important theoretical perspective for midwifery research. Midwifery. 2014;30(1):e1-6.

For peer review only

35. Connelly J. A realistic theory of health sector management: The case for critical realism J Manage Med. 2000;14(5/6):262-71.

36. Thomas P. General Medical Practitioners Need to Be Aware of the Theories on Which Our Work Depends. Ann Fam Med. 2006;4(5):450-4.

37. Akard T, Gilmer MJ, Friedman DL et al. From Qualitative Work to Intervention Development in Pediatric Oncology Palliative Care Research. J Pediatr Onc Nurs. 2013;30(3):153-60.

38. Kendall M, Harris F, Boyd K, et al. Key challenges and ways forward in researching the "good death": qualitative in-depth interview and focus group study. Br Med J. 2007;334(7592):521.

39. Murray SA, Kendall M, Carduff E, et al. Use of serial qualitative interviews to understand patients' evolving experiences and needs. Br Med J. 2009;339.

40. Gill P, Stewart K, Treasure E, et al. Methods of data collection in qualitative research: interviews and focus groups. Br Dent J. 2008;204(6):291-5.

41. Office for National Statistics. 2011 Census Glossary of Terms Office for National Statistics, 2014.

42. Carter B, Coad J, Goodenough T. Community Children's Nursing in England: An appreciative review of CCNs. Department of Health Report. NHS at Home: Community Children's Nursing Services. http://www.dh.gov.uk/en/Publicationsandstatistics/Publications/PublicationsPolicyAndGuidance/DH_1248-98.: 2009.

43. Greig A, Taylor J, MacKay T. Doing Research with Children. A practical guide. London: SAGE; 2008. p. 113-20.

44. Coad J, Gibson F, Horstman M, et al. Be my guest! Challenges and practical solutions of undertaking interviews with children in the home setting. J Child Health Care. 2015;19(4):432-43.

45. Lieblich A, Tuval-Mashiach R, Zilber T. Narrative research: reading, analysis and interpretation. London: Sage; 1998.

46. Bingley A, Thomas C, Brown J, et al. Developing narrative research in supportive and palliative care: the focus on illness narratives. Palliat Med. 2008;22(5):653-8.

47. Burnard P, Gill P, Stewart K, et al. Analysing and presenting qualitative data. Br Dent J. 2008;204(8):429-32.

48. Burnard P. A method of analysing interview transcripts in qualitative research. Nurs EdToday. 1991;11:461 - 6.

49. Kendall M, Murray SA, Carduff E, et al. Use of multiperspective qualitative interviews to understand patients' and carers' beliefs, experiences, and needs. Br Med J. 2009;339.

50. Staniszewska S, Boardman F, Gunn L, et al. The Warwick Patient Experiences Framework: patient based evidence in clinical guidelines. Int J Qual Health Care. 2014;26(2):151-7.

51. Medical Research Council. MRC Ethics Guide: Medical research involving children. London. www.mrc.ac.uk: Medical Research Council. , 2004.

52. Shaw C, Brady L-M, Davey C. Guidelines for Research with Children and Young People. London: National Children's Bureau, 2011.

53. Calman L, Brunton L, Molassiotis A. Developing longitudinal qualitative designs: lessons learned and recommendations for health services research. BMC Med Res Method. 2013;13(1):1-10.

For peer review only - http://bmjopen.bmj.com/site/about/guidelines.xhtml

54. Hui D, De La Cruz M, Mori M, et al. Concepts and definitions for "supportive care," "best supportive care," "palliative care," and "hospice care" in the published literature, dictionaries, and textbooks. Support Care in Canc. 2013;21(3):659-85.

55. Pastrana T, Ostgarthe C, Elsner F, et al. A matter of definition - key elements identified in a discourse analysis of definitions of palliative care. Palliat Med. 2008;22:222-32.

56. Twamley K, Craig F, Kelly P, et al. Underlying barriers to referral to paediatric palliative care services: knowledge and attitudes of health care professionals in a paediatric tertiary care centre in the United Kingdom. J Child Health Care. 2014;18(1):19-30.

57. Monterosso L, Kristjanson L. Supportive and palliative care needs of families of children who die from cancer: an Australian study. Palliat Med. 2008;22(1):59-69.

58. Morstad Boldt A, Yusuf F, Himelstein BP. Perceptions of the term palliative care. J Palliat Med. 2006;9(5):1128-36.

59. Crocker JC, Beecham E, Kelly P et al. Inviting parents to take part in paediatric palliative care research: A mixed-methods examination of selection bias. Palliat Med. 2015;29(3):231-40.

60. Hunt A, Coad, J, West, E, et al. The Big Study for Life-limited Children and their Families: Final research report. Bristol: Together for Short Lives, 2013.

For peer review only

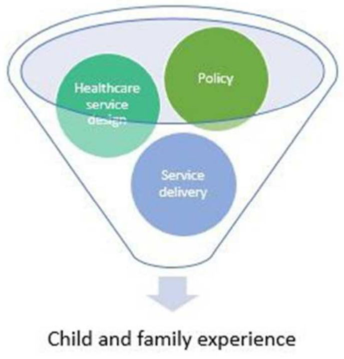

Figure 1: Healthcare policy, service design and delivery and their influence on the child and family experience as a causal intellectual puzzle.

61x28mm (300 x 300 DPI)

For peer review only

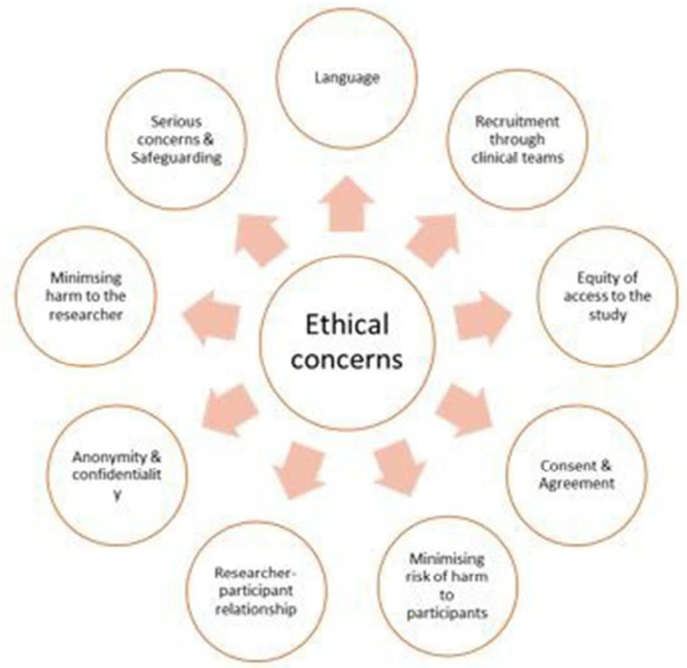

Figure 2: Ethical issues in longitudinal qualitative research for children and families in palliative care

36x31mm (300 x 300 DPI)

BMJ Editors Checklist for Qualitative Research

Checklist item	Page No
Was the research question clearly defined?	5
Overall, did the researcher make explicit in the account the theoretical framework and methods used at every stage or the research?	5,11
Was the context clearly described?	3,4,5
Was the sampling strategy clearly described and justified?<br>Was the sampling strategy theoretically comprehensive to ensure the generalisability of the conceptual analysis (diverse range of individuals and settings, for example)?	6
How was the fieldwork undertaken? Was it described in detail?	7-10
Could the evidence (fieldwork notes, interview transcripts, recordings, documentary analysis, etc) could be inspected independently by others: if relevant, could the process of transcription be independently inspected?	15, 16
Were the procedures for data analysis clearly described and theoretically justified? Did they relate to the original research questions? How were themes and concepts identified from the data?	11
Was the analysis repeated by more than one researcher to ensure reliability?	11
Did the investigator make use of quantitative evidence to test qualitative conclusions where appropriate?	N/A
Did the investigator give evidence of seeking out observations that might have contradicted or modified the analysis?	7,8,12,13,14
Was sufficient of the original evidence presented systematically in the written account to satisfy the sceptical reader of the relation between the interpretation and the evidence (for example, were quotations numbered and sources given)?	N/A

Consolidated criteria for reporting qualitative studies (COREQ): 32-item checklist

Developed from:
Tong A, Sainsbury P, Craig J. Consolidated criteria for reporting qualitative research (COREQ): a 32-item checklist for interviews and focus groups. *International Journal for Quality in Health Care*. 2007. Volume 19, Number 6: pp. 349 – 357

YOU MUST PROVIDE A RESPONSE FOR ALL ITEMS. ENTER N/A IF NOT APPLICABLE

No. Item	Guide questions/description	Reported on Page #
**Domain 1: Research team and reflexivity**		
Personal Characteristics		
1. Inter viewer/facilitator	Which author/s conducted the inter view or focus group?	1
2. Credentials	What were the researcher's credentials? E.g. PhD, MD	1
3. Occupation	What was their occupation at the time of the study?	1
4. Gender	Was the researcher male or female?	1
5. Experience and training	What experience or training did the researcher have?	8
Relationship with participants		
6. Relationship established	Was a relationship established prior to study commencement?	7
7. Participant knowledge of the interviewer	What did the participants know about the researcher? e.g. personal goals, reasons for doing the research	7
8. Interviewer characteristics	What characteristics were reported about the inter viewer/facilitator? e.g. Bias, assumptions, reasons and interests in the research topic	13-14
**Domain 2: study design**		
Theoretical framework		
9. Methodological orientation and Theory	What methodological orientation was stated to underpin the study? e.g. grounded theory, discourse analysis, ethnography, phenomenology, content analysis	6-7
Participant selection		
10. Sampling	How were participants selected? e.g. purposive, convenience, consecutive, snowball	7
11. Method of approach	How were participants approached? e.g. face-to-face, telephone, mail, email	7
12. Sample size	How many participants were in the study?	11

For peer review only

13. Non-participation	How many people refused to participate or dropped out? Reasons?	N/A
Setting		
14. Setting of data collection	Where was the data collected? e.g. home, clinic, workplace	8-9
15. Presence of non-participants	Was anyone else present besides the participants and researchers?	9
16. Description of sample	What are the important characteristics of the sample? e.g. demographic data, date	7
Data collection		
17. Interview guide	Were questions, prompts, guides provided by the authors? Was it pilot tested?	9-10
18. Repeat interviews	Were repeat inter views carried out? If yes, how many?	11
19. Audio/visual recording	Did the research use audio or visual recording to collect the data?	11
20. Field notes	Were field notes made during and/or after the inter view or focus group?	11
21. Duration	What was the duration of the inter views or focus group?	N/A
22. Data saturation	Was data saturation discussed?	11
23. Transcripts returned	Were transcripts returned to participants for comment and/or correction?	12
**Domain 3: analysis and findings**		
Data analysis		
24. Number of data coders	How many data coders coded the data?	12
25. Description of the coding tree	Did authors provide a description of the coding tree?	12
26. Derivation of themes	Were themes identified in advance or derived from the data?	12
27. Software	What software, if applicable, was used to manage the data?	12
28. Participant checking	Did participants provide feedback on the findings?	N/A
Reporting		
29. Quotations presented	Were participant quotations presented to illustrate the themes/findings? Was each quotation identified? e.g. participant number	N/A
30. Data and findings consistent	Was there consistency between the data presented and the findings?	N/A
31. Clarity of major themes	Were major themes clearly presented in the findings?	N/A
32. Clarity of minor themes	Is there a description of diverse cases or discussion of minor themes?	N/A

Once you have completed this checklist, please save a copy and upload it as part of your submission. When requested to do so as part of the upload process, please select the file type: *Checklist*. You will NOT be able to proceed with

For peer review only

submission unless the checklist has been uploaded. Please DO NOT include this checklist as part of the main manuscript document. It must be uploaded as a separate file.

For peer review only

BMJ Open

The Journey through Care: Study protocol for a longitudinal qualitative interview study to investigate the healthcare experiences and preferences of children and young people with life-limiting and life-threatening conditions, and their families in the West Midlands, UK.

For peer review only

Journal:	*BMJ Open*
Manuscript ID	bmjopen-2017-018266.R2
Article Type:	Protocol
Date Submitted by the Author:	11-Oct-2017
Complete List of Authors:	Mitchell, Sarah; Warwick Medical School<br>Slowther, Anne-Marie; University of Warwick, Warwick Medical School<br>Coad, Jane; Coventry University<br>Dale, Jeremy; University of Warwick, Warwick Medical School
**Primary Subject Heading**:	Palliative care
Secondary Subject Heading:	Health services research, Paediatrics, Patient-centred medicine, Qualitative research
Keywords:	Organisation of health services < HEALTH SERVICES ADMINISTRATION & MANAGEMENT, Paediatric palliative care < PAEDIATRICS, PALLIATIVE CARE, QUALITATIVE RESEARCH

SCHOLARONE™
Manuscripts

The Journey through Care: Study protocol for a longitudinal qualitative interview study to investigate the healthcare experiences and preferences of children and young people with life-limiting and life-threatening conditions, and their families in the West Midlands, UK.

Mitchell S[1], Slowther A-M[1], Coad J[2], Dale J[1].

Corresponding Author: Sarah Mitchell MB ChB FRCGP MSc

National Institute of Health Research Doctoral Research Fellow

Sarah.j.mitchell@warwick.ac.uk; 07950202240; @MacGPSarah

Author affiliations:

1. Warwick Medical School, University of Warwick, Gibbet Hill Road, Coventry, CV4 7AL
2. Faculty of Health and Life Sciences, Coventry University, Priory Street, Coventry, CV1 5FB.

Word counts

- Abstract: 461
- Protocol: 4183

Tables: 5

Boxes: 1

Colour Figures: 1

For peer review only

Abstract

INTRODUCTION

The numbers of children and young people living with life-limiting and life-threatening conditions is rising. Providing high quality, responsive healthcare for them and for their families presents a significant challenge. Their conditions are often complex and highly unpredictable. Palliative care is advocated for people with life-limiting and life-threatening conditions, but these services for children are highly variable in terms of availability and scope. Little is known about the lived experiences and preferences of children and their families in terms of the palliative care that they do, or do not, receive.

This study aims to produce an in-depth insight into the experiences and preferences of such children and families in order to develop recommendations for the future provision of services. The study will be carried out in the West Midlands, UK.

METHODS AND ANALYSIS

A qualitative study comprising longitudinal interviews over a 12 month period with children (aged 5-18 years) living with life-limiting or life-threatening conditions, their family members.

Data analysis will start with thematic analysis, followed by narrative and cross-case analysis to examine changing experiences and preferences over time, at the family level and within the wider healthcare system.

Patient and Public Involvement (PPI) has informed the design and conduct of the study. Findings will be used to develop recommendations for an integrated model of palliative care for children in partnership with the PPI group.

ETHICS AND DISSEMINATION

Ethical approval was granted in September 2016 by the NHS Health Research Authority (IRAS ID: 196816, REC reference: 16/WM/0272).

Findings will be of immediate relevance to health care providers, policy makers, commissioners and voluntary sector organisations in the UK and internationally. Reports will be prepared for these audiences, as well as for children and their families, alongside academic outputs.

STRENGTHS AND LIMITATIONS

- An in-depth, contextual, longitudinal qualitative study with multiple child and family member stories captured over time.

For peer review only

- New insights will be provided because all of the children and families included in the study could benefit from palliative care as it is currently defined, however not all will have had conversations about this or have been referred to specialist palliative care services. Findings will focus on healthcare, but there is wider applicability and relevance to social care and joint planning of services.
- A diverse study population in terms of age, clinical condition, cultural background and family structure will allow detailed consideration of the role of healthcare services in effectively recognising and supporting children and families with their individual needs. However all will speak English.
- Neonates, pre-school children and young people at transition (over the age of 18 years) are all excluded and warrant research in their own right.
- There are multiple potential sources of bias which will be addressed throughout the study, including recruitment bias and the unconscious bias of the researcher.

Author's contributions

SM drafted the protocol with regular academic supervision from JD, A-M S and JC. The study was conceptualised by SM, JD, A-MS and JC, informed and guided by Patient and Public Involvement. The protocol incorporates peer review feedback received during the application process for the NIHR Doctoral Research Fellowship, and the MPhil to PhD upgrade panel at the University of Warwick. JD, A-MS and JC have all reviewed the protocol for intellectual content. All authors have reviewed and agreed this version.

Funding statement

This work is supported by a National Institute of Health Research Doctoral Research Fellowship (Dr Sarah Mitchell DRF-2014-07-065)

Competing interests

The authors have no competing interests.

For peer review only

Introduction

Children and young people with life-limiting conditions and life-threatening conditions represent a growing concern in healthcare (1). With advances in clinical practice, the numbers of children living with these conditions is rising (1-3). The nature of their conditions is complex and unpredictable; the risk of a sudden deterioration and death is an everyday reality for many. Family carers can experience enormous emotional, physical and financial pressures (4).

Research suggests that families of children with life-limiting and life-threatening conditions wish for continuous, holistic healthcare, with the option that this is delivered in the home environment (4). However most children who die have a pre-existing life-limiting condition (5), and most die in hospital (6, 7), most frequently in an intensive care environment, where the mode of death is often withdrawal or limitation of life-sustaining treatments (8-10). The length of stay in the intensive care unit before death is increasing , and the costs of hospital care at the end of life are significant (11, 12). This situation presents complex clinical and ethical dilemmas at individual, organizational and societal levels.

Palliative care is an approach to care that is advocated for all people who live with a life-limiting or life-threatening condition. Current definitions of palliative care are broad (box 1), which can cause difficulties and lack of clarity for those designing and commissioning specific services.

Box 1: Children's Palliative Care Definitions (13, 14):

Palliative care for children with life-threatening conditions is defined by the World Health Organisation as "a special, albeit closely related field to adult palliative care; the principles apply to other paediatric chronic disorders:

- The active total care of the child's body, mind and spirit, and support to the family.
- It begins when illness is diagnosed, and continues regardless of whether or not a child receives treatment directed at the disease.
- Health providers must evaluate and alleviate a child's physical, psychological, and social distress.
- Effective palliative care requires a broad multidisciplinary approach that includes the family and makes use of available community resources; it can be successfully implemented even if resources are limited.
- It can be provided in tertiary care facilities, in community health centres and even in children's homes"

The UK national charity for paediatric palliative care, Together for Short Lives, defines palliative care for children with life-limiting conditions as "an active and total approach to care, from the point of diagnosis or recognition, embracing physical, emotional, social and spiritual elements through to death and beyond. It focuses on enhancement of quality of life for the child and support for the family and includes the management of distressing symptoms, provision of short breaks and care through death and bereavement".

For peer review only - http://bmjopen.bmj.com/site/about/guidelines.xhtml

For clarity, in this paper children and young people will be referred to throughout as “children”.

WHICH CHILDREN?

There has been a range of previous work concerned with the identification of clinical conditions where palliative care could be beneficial (15, 16), and the following categorisation is provided by Together for Short Lives (14):

- *Group 1:* life-threatening conditions where access to palliative care services is necessary alongside attempts at curative treatment and / or if treatment fails, such as cancer.
- *Group 2:* conditions such as Duchenne muscular dystrophy, where premature death is inevitable, but where there may be long periods where the child is well.
- *Group 3:* progressive conditions without curative treatment options, such as Batten disease.
- *Group 4:* irreversible but non-progressive conditions, with complex disabilities and healthcare needs which lead to increased likelihood of premature death, such as severe brain injury.

ORGANISATIONAL ISSUES

Currently there is wide geographic variation in terms of paediatric palliative care services, and a poorly understood range of commissioning arrangements to support these services. Many services exist as a result of significant contributions from the voluntary sector (including children’s hospices), through the efforts of motivated individuals, and through non-recurring funding opportunities rather than the implementation of national policy (17). A significant development is the emergence of paediatric palliative medicine as a sub-speciality of paediatrics (18, 19).

Effective palliative care services for children require strong partnerships between providers, and may require cross-boundary, collaborative commissioning between the statutory and voluntary sectors (20). In the UK, palliative care for CYP has specifically been included in national policy , a service specification for paediatric palliative care exists and NICE (National Institute for Health and Care Excellence) Guidelines for End-of-Life Care for Infants, Children and Young People were published in 2016 (21-25).

ONTOLOGY, EPISTEMOLOGY AND THEORETICAL PERSPECTIVE

Much of the evidence base that guides policy and practice in medicine is derived from experimental research grounded in a positivist paradigm, for example randomised controlled trials, where a hypothesis can be generated and tested. The positivist approach does not lend itself to research which aims to investigate more complex interventions, such as palliative care, and an interpretive approach is more appropriate. The experience of health care services by children with a life-limiting

For peer review only

or life-threatening condition and members of their family are shaped and influenced by many interlinked factors including their own personal experiences, values, and cultural influences, the values of the health care team and the health care system, the specific context in which care is delivered and the relationships between those involved in providing and receiving care (26-28).

The proposed research seeks to understand the mechanisms and influences that shape the experience of care in order to inform both the development and implementation of policy for palliative care services for young children with life limiting conditions. The methodological approach identified as most appropriate for this research aim was critical realism, an approach which is increasingly used in healthcare and health sector management research (29-32). First described by Bhaskar in the 1970s (33), critical realism seeks to understand how phenomena come about as a result of hidden mechanisms, enacted under certain circumstances (34, 35). It acknowledges that there are a wide variety of dynamic contexts and mechanisms which can affect outcomes, including geographical and environmental factors, social and cultural issues and historical factors, and provides a generative approach allowing for the proposal of theories to guide the implementation of policy into practice (36).

RATIONALE FOR RESEARCH

Despite the range of recommendations for the provision and development of paediatric palliative care services, there remains a lack of research evidence to support the implementation of these guidelines (1, 19, 37-39). The proposed research seeks to address this gap using a critical realist approach to address research questions that correlate with the practical concerns associated with service delivery. The findings and theories that are generated will provide in-depth insights that will be of immediate relevance to clinicians, commissioners and policy makers, as well as to patients and their families.

RESEARCH QUESTIONS

1. How do children with life-limiting and life-threatening conditions and their family members perceive healthcare services, and in particular “palliative care”?
2. What are the experiences and preferences of children living with a life-limiting or life-threatening condition, and/or their families, in relation to the delivery of healthcare services?
3. What are the facilitators and barriers to the delivery of palliative care for children, and how might these be overcome?
4. What should an integrated model of palliative care for children look like?

For peer review only

Methods and Analysis

In order to conduct an exploration of the experiences of healthcare from the perspective of children with life-limiting and life-threatening conditions and their families, we will adopt qualitative research methods and a narrative-based approach, suitable for complex, emotionally charged subject areas (40, 41). Active listening, reflection, a flexible approach and insight into the narratives being co-constructed between participant and researcher will be necessary throughout.

This is the protocol for an in-depth longitudinal qualitative study using semi-structured interviews with school-aged children (5-18 years) and one or two of their household family members.

Benefits of longitudinal studies include being able to describe the changing needs of the children and their families, and their experience of services, over time (42), and enabling rapport to build between researcher and participant.

Neonates, pre-school children and young people aged over the age of 18 years are excluded from this study. Specific issues around healthcare services arise when considering neonatal care and young people who are making the transition from paediatric to adult services, both of which warrant research in their own right. Research methods would need to be tailored to interview pre-school children; this is also an area for potential future research.

The research plan has been informed by review of relevant literature, patient and public involvement (PPI) work and advice from local experts via the West Midlands Paediatric Palliative Care Network.

SAMPLING AND RECRUITMENT

Recruitment to a study of this nature depends on many factors, including the clinical condition of the child, conflicting demands on the family's time, and the motivation and understanding of their clinical teams. Recruitment began following ethical approval in October 2016 and will continue until January 2018.

The approach to participants is through:

1. direct invitation via their clinical team
2. leaflets and posters displayed in public areas in the hospital (such as notice boards on wards and in outpatients).

The research will be introduced to clinical teams in both the hospital and the community through formal presentations at departmental meetings and to individual clinicians at their request, as well

For peer review only

as to the paediatric palliative care network. The researcher, SM, will undertake a period of shadowing with clinical teams, on hospital wards, in outpatient clinics and in the community.

Potential participants will be provided with a participant information sheet, with details of the researcher, the project, how to get involved, and a contact telephone number and email address.

Inclusion and exclusion criteria are outlined in Table 1. Our aim initially is to purposively sample children so that each of the four Together for Short Lives categories are represented. However, since children live with such individual and highly complex conditions we anticipate that achieving this may be difficult. The study population will therefore be children with life-limiting or life-threatening conditions, aged from 5-18 years, and their family members, some of whom have experience of a palliative care service, and some who do not.

The study has been carefully designed to ensure that all of the children have the opportunity to participate and that wherever possible the views of the child are included, by tailoring each individual interview to their needs and capabilities (including the consent and agreement process). This may include having a learning disability or communication difficulties associated with their condition.

Ethical approval has been granted for the recruitment of 12-14 families to take part in a series of interviews (longitudinal interviews). The aim is to continue to conduct interviews until data saturation is achieved (43), however given the uniqueness and individuality of the stories of children and families, it is possible that new themes will continue to emerge such that data saturation is impossible. We will aim for saturation of the main themes that emerge from the data, and identify emergent themes, which may form the basis of future research.

Table 1: Inclusion and Exclusion Criteria

Inclusion Criteria	1. Children aged 5-18 yrs (school age) with a life-limiting or life-threatening condition who are under the care of the Community Children's Nursing Team and/or the Children's Hospital and who either:<br>• receive palliative care services<br>• are aware of (have had discussions about) palliative care services<br>• are living with relapsing or refractory disease<br>• or have had a life-threatening episode (admission to the Paediatric Intensive Care Unit (PICU))<br><br>2. Their family members, who live in the same household (44).

For peer review only - http://bmjopen.bmj.com/site/about/guidelines.xhtml

Exclusion Criteria	• Children aged < 5 years and > 18 years.<br>• Families of children < 5 years and > 18 years old<br>• Children and families with whom the research team has clinical contact<br>• Children and / or families who do not wish to participate.<br>• Children who are too unwell will not be approached for interview, but their family members may still participate if they wish to. The researcher will take advice from parents about when an individual child is "too unwell" to take part.<br>• Children who are unable to participate in a conversational interview for any reason related to their condition will not be approached for interview, but their family members may participate if they wish to.<br>• Children and families who are unable to provide informed consent in English will not be approached for interview.

INTERVIEW PLAN

Interviews will be carried out by SM, a researcher who is also a GP with advanced communication skills training and previous experience in qualitative interviewing. According to the preference of participants, interviews will be conducted with individuals, or with the child and parent together, in their preferred location. One or two family members will be interviewed in each family, either individually or together, depending on their preference and what is most convenient for them.

Interviews will be open and conversational, using a blended approach of interview techniques, with passive interviewing allowing the participant space and time to tell their story (narrative), and more active techniques, including appreciative inquiry, which asks "what works well?" and "why does it work well?" (4, 45), employed. A topic guide (table 2) will guide the interview; this will continue to develop iteratively throughout the research, with adaptations made during each interview and in response to each individual participant.

Table 2: Topic Guide

For all families	For those aware of "palliative care"
**Introduction**<br><br>**Please tell me your story, in any way that you can / want to**<br><br>**Please tell me the story of you**	*Palliative care and you (if appriopriate)*<br><br>**Do you have "palliative care" services?**<br><br>Have you ever heard the term "palliative care"?

For peer review only

Your Story<br><br>**Please can you tell me a about you?**<br><br>**Your family?**<br><br>**Your child(ren)**<br><br>**What is important to you?**<br><br>**What do you like to do?**<br><br>**Which places are important to you?**<br><br>**Where do you spend your time?**<br><br>**Which services are involved in your care?**<br><br>**Who comes to see you?**<br><br>**What do they do?**<br><br>What's helpful?<br><br>What's not?<br><br>**Which healthcare professionals do you consider to be key in the delivery of your care?**<br><br>**What works best?**<br><br>Which services / professionals are most helpful?<br><br>Which services / professionals do you value most?<br><br>**What doesn't work?**<br><br>**How do you think services could be improved?**	What does that mean to you?<br><br>What do you receive those services for?<br>What do these services provide for you?<br>Does it matter what a service is called?<br><br>Do you receive services from the hospice?<br><br>**Can you tell me how you came to receive palliative care / know the palliative care nursing team / the hospice?**<br><br>When were you referred?<br><br>Who brought it up / made the referral?<br><br>How was this discussed with you?<br><br>How was that for you / your family?<br><br>**Do you think that medical / nursing staff receive enough training in this area?**<br><br>What makes you think that?<br><br>**Anything else?**

For peer review only

**Do you talk to other children / young people / families about your healthcare / services"?**<br><br>What do you tell your friends?<br><br>What tends to come up in these discussions?<br><br>Would you recommend these services to others?	

For interviews with children, a range of techniques will be used including de-personalising questions, developing a narrative in the third person, and using props and toys to encourage story-telling. Arts-based activities will be used, where appropriate, as a mutual point of focus for the researcher and participant, or as a focus of the interview, as in the draw-write-tell technique (46). PPI advice has been sought on the format of interviews for children (Table 3).

Table 3: Feedback from PPI groups on interview plans

January 2016	*"Those who are passionate about improving palliative care will take part regardless of how sensitive this may be"*
July 2016	*"Remember young people who are seriously ill are more mature, they have to grow up"*<br><br>*"Keep it simple as often a child will openly speak anyway"*<br><br>*"'Do you talk about it to your friends?' is a good question, a good way to talk to most ages."*<br><br>*"Children are more eloquent, mature and more capable than you think"*
October 2016	*"Use pictures and images, more emojis"*
February 2017	*"Doesn't make me uncomfortable as I think it is very important and relevant"*

Each interview will be audio-recorded, with field notes made to include any additional comments from the child or family made once the audio-recording has stopped (47), reflections on the interview and observation of the family situation, environment, behaviour, and any other

For peer review only - http://bmjopen.bmj.com/site/about/guidelines.xhtml

interactions that may take place (for example with other members of the family and clinical staff in hospital or on the phone).

Participants will be asked whether they would like to participate in interviews that will take place over a period of up to 12 months. These are intended to allow the identification of common themes over time and for theories generated through analysis of earlier interviews to be tested out during later interviews (42). The time intervals between interviews will be individually agreed, depending on the child and family circumstances. The method of communication with each family will also be individually agreed (phone or email). Up to three interviews with each participant are aimed for.

We anticipate practical challenges with conducting longitudinal interviews relating to fluctuations and changes in the clinical condition of each child. Depending on their condition, some children will respond well to treatments and get better. Others may suffer unexpected complications of their condition or treatment, and some may suffer deteriorations which bring about the possibility of dying. To manage the research in this context, we will check the family understanding of the situation before every interview. On occasions, interviews may need to be postponed and rearranged at late notice due to a change in circumstances.

For children who are unable to participate in interviews due to their condition, family members will be interviewed. Children and families are under no obligation to take part in follow-up interviews if they do not wish to. In these cases, and with their consent, data from previous interviews will still be included in the study.

DATA ANALYSIS

Interviews will be transcribed verbatim, and NVivo used for data handling. Analysis of interview transcripts and field notes will commence alongside data collection, with an initial broad thematic analysis. All data will be coded, and codes grouped into broad overarching themes.

This initial analysis will be followed by an in-depth, narrative analysis, using structure-form analysis to examine not just what is being said, but how it is being said, and to position narratives in the context of palliative care from a micro (immediate clinical team), meso (local organisation) and macro (wider healthcare system) level perspective (48, 49).

The collection of longitudinal data allows for innovative approaches to be taken in data analysis (42). Matrices will be developed to identify key times for families and identification of cross-cutting themes at these times, for example the time of diagnosis, an admission to intensive care or referral to a palliative care team.

Peer review and respondent validation will take place throughout the data analysis as follows (50, 51):

For peer review only

1. Peer review: SM will code all of the data. A selection of transcripts will be reviewed and independently coded by other members of the research team in order to decrease lone researcher bias (50). The coding frameworks will be discussed and compared, allowing further development of categories and themes.
2. Respondent validation: By returning to participants to conduct longitudinal interviews, there is opportunity to check, validate or refute emergent themes from the initial data analysis.

HEALTHCARE PROFESSIONAL PERSPECTIVES

There are 12 paediatric palliative care networks in the UK, which include professionals from a range of organisations within paediatric palliative care. Several have patient and family representatives. Arrangements will be made to present study findings to four of the UK networks at existing meetings. The presentation will be followed by a structured focus group which will aim to firstly to test out and validate with palliative care professionals the themes from the research findings, and secondly aims to collect views of professionals. These multiple perspectives will inform and guide the formulation of recommendations for healthcare services in the future (52).

An expression of interest email will be circulated to network chairs via Together for Short Lives, and arrangements made to attend meetings from networks who respond. Audio-recorded focus group discussions will be carried out at those meetings by SM.

Patient and Public Involvement

PPI has been integral to the design and conduct of the study. Members of existing groups at a children's hospital and children's hospice have provided advice on the study proposal and design. Smaller groups have been recruited for specific activities, including conference presentations. Group members range in age from 12 to 22 years. PPI activities are outlined in Table 4, and will continue throughout the project, with the aim of co-producing the recommendations for the model of care. This will involve structured group sessions during which anonymised findings of the data analysis will be presented to the group for feedback and comment. A patient experience framework will be used to structure the discussion and to build recommendations (53).

Table 4: PPI activities

Completed PPI activities	• Developing the original research proposal<br>• Advising on the language used in the study (suggesting a change in the title from "Palliative Care for Children and Young People: What? When? How?" to "The Journey

For peer review only - http://bmjopen.bmj.com/site/about/guidelines.xhtml

For peer review only

	through Care".<br>• Developing participant resources including leaflets for older and younger children<br>• Interview design, including suggesting how questions could be phrased and asked<br>• Providing family perspectives to a literature review, and becoming a co-author on the paper (19).<br>• Taking part in oral presentations at regional conferences
Work in progress	• Designing conference posters and presentations for national conferences<br>• Working as co-researchers to carry out a survey study to investigate understanding of the term "palliative care" for children and young people, and healthcare professionals
Future plans	• Working with a project-specific group to explore the findings of the research study and develop recommendations for a new model of care.<br>• Dissemination projects including conference presentations, posters, website design, use of social media, infographics and films.

Strengths and Limitations

The strength of this study lies in the in-depth, contextual qualitative nature of the data, with multiple child and family member stories captured over time. Our anticipated study population is diverse in terms of age, clinical condition, cultural background and family structure, allowing detailed consideration of the role of healthcare services in effectively recognising and supporting children and families with their individual needs. All of the children and families included in the study could benefit from palliative care as it is currently defined (14), however not all will have had conversations about this with their clinicians, or been referred to specialist palliative care services. Given the nature of their clinical conditions, including for some the inability to communicate verbally or deterioration in their health, recruitment and retention within the study is likely to become a challenge and will require a reflexive, flexible approach.

Potential limitations in the study include our exclusion of neonates, pre-school children and young people at transition (over the age of 18 years). These groups all warrant research in their own right. Given the time and resource constraints of the study, all interviews will be carried out in English. Further research into the experiences of CYP and families who cannot communicate in English is necessary. There will be ongoing consideration of sources of bias. Recruitment bias is being addressed by aiming for a diverse sample and providing access to project information independent

For peer review only

of the clinical teams. Data saturation will be sought during data analysis, with an ongoing process of reflection and peer review to address any possible unconscious bias of the researcher (SM).

Ethics and Dissemination

Research with children raises ethical and legal considerations around recruitment, consent, and data collection (54, 55). In addition research regarding palliative and end-of-life care can be emotionally demanding and distressing for those involved. There are also particular ethical issues to consider given the longitudinal nature of the study(56).

We are recruiting children and families who are potentially vulnerable and may be experiencing considerable distress. The justification for our approach is that children and their families in this situation are rarely asked about their experiences, but talking to them and understanding their experiences is essential in order to be able to design and develop services that respond to their actual needs. Here we summarise our approach to the ethical issues the study raises (Figure 1).

Figure 1: Ethical issues in longitudinal qualitative research for children and families in palliative care

LANGUAGE

Published literature suggests that the term "palliative care" is poorly understood and perceived negatively (57-61), a view confirmed by our PPI group. The scope of our study is therefore to investigate the experiences of children with "life-limiting", "life-threatening" and "conditions which may or may not get better", whether or not they have heard of palliative care or receive care from specialist services. "Palliative care" will be avoided in participant information sheets and interviews, unless individuals are already familiar with palliative care services or bring it up themselves.

RECRUITMENT

There is an ethical challenge in terms of potential coercion to the study by clinicians who know the family well. Clinicians will therefore only provide study information but will not actively recruit families; the initial expression of interest is from the family to the researcher. The researcher (SM) will then discuss the study in person or by phone with the child and their family member(s) and answer any questions before arranging a time for interview. Participants will be made aware that they can decline to take part or to withdraw at any stage without having to give a reason. Interviews are only carried out at a time that is mutually agreed and minimises any potential inconvenience or intrusion.

For peer review only

Equity of access to the study

Recruitment through clinical teams is widely used in palliative care research but may be limited by "gatekeeping" (62). There may also be families who wish to participate who do not find out about the study through their clinical team. In order to address this, we have designed posters for display on hospital wards and in outpatients, and at the local children's hospice, and a paragraph for organisational newsletters. These provide the direct contact details for the researcher (email, text or phone).

The study setting is Birmingham, UK, a city where the population is highly diverse in terms of family situation and multiculturalism. Over 50% of families with a child known to palliative care services in Birmingham and Solihull are from black or minority ethnic backgrounds (63). Many of these families speak English as a first or second language , so within the time and resource constraints of this study, interviews will be carried out with those who can provide informed consent and take part in an interview in English.

CONSENT

Consent for the study raises ethical and legal issues with children who are under the age of 16 years and / or do not have the capacity to consent. We will aim for written and / or verbal consent and agreement from every individual for every interview.

For children under the age of 16, written consent will be obtained from the parent and then verbal or written agreement obtained from the child.

In keeping with the Mental Capacity Act, there is an assumption of capacity in young people aged 16 years and over, so they will be asked for consent first, followed by agreement from their parent(s). Parental agreement is not a legal requirement, but conducting an interview with a young person about a potentially difficult subject without the knowledge or agreement of their parents is an ethical concern. If there is a concern that the young person lacks capacity or is considered particularly vulnerable, for example with a learning disability, parents will be asked to provide verbal and written consent in addition to the young person's agreement.

Parental consent is required for any interview to be carried out in the family home (55).

For a child on a full care order, social worker consent would replace that of parental consent. Where possible parental consent / agreement will also be sought.

For peer review only

INTERVIEWS

Subject areas discussed during interviews may cause distress to participants, and recruitment may occur soon after sensitive conversations. We have designed the study to ensure that the risks and burden associated with taking part in the study are minimal.

Qualitative interviews will be informal and reflexive to the needs of the participant. In the event that a participant experiences any difficulties during the interview, such as tiredness or distress, the interview will be halted, and if necessary brought to an end. Adequate time will be given for debrief, and the researcher will provide information about local resources for support if necessary. Interviews will be carried out at a time and in a location that is convenient to the individual participant. If this is in hospital, the researcher (SM) will liaise closely with clinical teams so that the research does not interfere with routine clinical care and ward work.

Longitudinal interviews

Family views and understanding of what might happen next as a result of the condition of the child will be discussed sensitively, and any follow-up interviews scheduled around possible further treatments. If it seems likely that there will be a deterioration in the condition of the child, this is explored carefully and an agreement made with the individual family about whether they want to continue to participate in the study.

ANONYMITY AND CONFIDENTIALITY

All interview data will be anonymised with personal identifiers removed. Any qualitative interview data that could identify child, families or any professionals involved in their care because of the individuality and context of the narrative is included in the data analysis, but will be excluded from reporting.

Field notes and anonymised interview transcripts will be stored securely on a password protected university hard drive.

MINIMISING HARM TO THE RESEARCHER

There is a need for clearly defined boundaries for a researcher – participant relationship in a longitudinal study of this type. It will be made clear to participants at the time of consent that it is not the role of the researcher to provide personal support or clinical advice. With the risk of emotional distress for the researcher, plans to ensure adequate support through regular academic supervision and access to a counsellor are in place.

SERIOUS CONCERNS AND SAFEGUARDING

If information contained in a participant's response indicates a serious clinical or safeguarding concern or an issue which may jeopardise the safety of the participant or another person, this will be

For peer review only

escalated appropriately in line with the protocols of the community or hospital trusts. This may on very rare occasions necessitate a breach of participant confidentiality in order to maintain their safety. Participants will be informed of any disclosure and to whom it is made.

ETHICAL APPROVAL

Ethical approval was granted by the UK Health Research Authority on 14th September 2016 (IRAS ID: 196816, REC reference: 16/WM/0272, Sponsor: University of Warwick).

DISSEMINATION PLAN

The research is embedded in plans for impact. Table 5 outlines our planned outputs. We will work on traditional academic and clinical outputs, including manuscripts with the results of the study for publication in a peer-reviewed journal. Simultaneously work will be carried out with the PPI group to plan innovative, accessible outputs for patients, the public and commissioners which will include infographics and film based reports outlining our recommendations.

Table 5: Planned outputs from the research

Academic / Clinical audiences	Patient, public and policy-maker audiences
• PhD Thesis<br>• Peer reviewed publications<br>• Presentations at national and international conferences	• A report prepared for participants and PPI volunteers<br>• The development of guidance for commissioners and providers<br>• The development of resources that are accessible to patients and families

For peer review only

References

1. Fraser LK, Miller M, Hain R, et al. Rising national prevalence of life-limiting conditions in children in England. Pediatrics. 2012;129(4):e923-9.
2. Fraser LK, Parslow RC, McKinney PA, et al. Life-limiting and life-threatening conditions in children and young people in the United Kingdom. Pediatr. 2012;129(4):1-7.
3. Office for National Statistics (2007). Mortality Statistics: deaths registered in 2007.
4. Coad J, Kaur J, Ashley N, et al. Exploring the perceived met and unmet need of life-limited children, young people and families. J Pediatr Nurs. 2015;30(1):45-53.
5. Sidebotham P, Fraser J, Fleming P, et al. Patterns of child death in England and Wales. The Lancet. 2014;384(9946):904-14.
6. Confidential Enquiry into Maternal and Child Health (2008). Why Children Die: a pilot study. London: CEMACH, 2008.
7. Department of Health (2007). Palliative Care Statistics for Children and Young Adults. London: Department of Health; 2007.
8. McCallum DE, Byrne P, Bruera E. How children die in hospital. J Pain Sympt Manage. 2000;20(6):417-23.
9. Ramnarayan P, Craig F, Petros A, et al. Characteristics of deaths occurring in hospitalised children: changing trends. J Med Ethics. 2007;33(5):255-60.
10. Epstein D, Brill JE. A history of pediatric critical care medicine. Pediatr Res. 2005;2005(58):987-96.
11. Widger K, Seow H, Rapoport A, Chalifoux M, Tanuseputro P. Children's End-of-Life Health Care Use and Cost. Pediatr. 2017.
12. Plunkett A, Parslow RC. Is it taking longer to die in paediatric intensive care in England and Wales? Arch Dis Child. 2016;101:798–802.
13. World Health Organisation. http://www.who.int/cancer/palliative/definition/en/.
14. Together for Short Lives. Children's Palliative Care Definitions. http://www.togetherforshortlives.org.uk/assets/0000/4089/CPC_definitions.pdf.
15. Hain R, Devins M, Hastings R, et al. Paediatric palliative care: development and pilot study of a 'Directory' of life-limiting conditions. BMC Palliat Care. 2013;12(1):43.
16. Noyes J, Edwards RT, Hastings RP, et al. Evidence-based planning and costing palliative care services for children: novel multi-method epidemiological and economic exemplar. BMC Palliat Care. 2013;12(1):18.
17. MacNamara-Goodger K, Feudtner C. History and epidemiology. In: Goldman A, Hain R, Liben S, Ed. Oxford Textbook of Palliative Care for Children 2nd ed. Oxford: Oxford University Press; 2012.
18. Hain R, Heckford E, McCulloch R. Paediatric palliative medicine in the UK: past, present, future. Arch Dis Child. 2012;97(4):381-4.
19. Mitchell S, Morris A, Bennett K, et al. Specialist paediatric palliative care services: what are the benefits? Arch Dis Child. doi: 10.1136/archdischild-2016-3120262017.
20. Rowse V. Home-based palliative care for children: the case for funding. Paediatric nursing. 2006;18(7):20-4.

For peer review only

21. National Institute for Health and Care Excellence (NICE). End of Life Care for Infants, Children and Young People. https://www.nice.org.uk/guidance/ng61

22. Villanueva G, Murphy MS, Vickers D, et al. End of life care for infants, children and young people with life limiting conditions: summary of NICE guidance. Br Med J. 2016;355.

23. Scottish Government. Strategic Framework for Action on Palliative and End of Life Care. 2015. http://www.gov.scot/Topics/Health/Quality-Improvement-Performance/peolc/SFA

24. Welsh Government. End of life care delivery plan. http://gov.wales/topics/health/nhswales/plans/end-of-life-care/?lang=en: 2014.

25. Department of Health, Social Services and Public Safety, Northern Ireland. Living Matters, Dying Matters: A palliative and end of life care strategy for adults in Northern Ireland www.dhsspsni.giv.uk/publications/living-matters-dying-matters-strategy-2010.

26. Mason J. Qualitative Researching. London: SAGE Publishing; 2002.

27. Crotty M. The Foundations of Social Research: Meaning and perspective in the research process. Sydney: Allen & Unwin; 1998.

28. Ormston R, Spencer L, Barnard M, et al. The Foundations of Qualitative Research. In: Ritchie J, Lewis J, , McNaughton Nicholls C, Ormston R Ed. Qualitative Research Practice. London: SAGE; 2013.

29. Schiller CJ. Critical realism in nursing: an emerging approach. Nurs Philos. 2016;17(2):88-102.

30. Walsh D, Evans K. Critical realism: an important theoretical perspective for midwifery research. Midwifery. 2014;30(1):e1-6.

31. Connelly J. A realistic theory of health sector management: The case for critical realism J Manage Med. 2000;14(5/6):262-71.

32. Ragin C, Becker H. What is a case? Exploring the foundations of social inquiry: Cambridge University Press; 1992.

33. Bhaskar R. A realist theory of science. . Brighton: Harvester; 1975.

34. Pawson R, Tilley N. Realistic evaluation. London: Sage Publications; 1997.

35. Clark AM, MacIntyre PD, Cruickshank J. A critical realist approach to understanding and evaluating heart health programmes. Health. 2007;11(4):513-39.

36. Sayer A. Realism and social science. . London: Sage Publications; 2000.

37. Hain RW. Palliative care in children in Wales: A study of provision and need. Palliat Med 2005;19(2):137-42.

38. Hain RW. Palliative care in children: Who needs it, and why ? Acta Paediatrica 2011;100:4.

39. Baker JN, Levine DR, Hinds PS, et al. Research Priorities in Pediatric Palliative Care. J Pediatr. 2015;167(2):467-70 e3.

40. Akard T, Gilmer MJ, Friedman DL et al. From Qualitative Work to Intervention Development in Pediatric Oncology Palliative Care Research. J Pediatr Onc Nurs. 2013;30(3):153-60.

41. Kendall M, Harris F, Boyd K, et al. Key challenges and ways forward in researching the "good death": qualitative in-depth interview and focus group study. Br Med J. 2007;334(7592):521.

42. Murray SA, Kendall M, Carduff E, et al. Use of serial qualitative interviews to understand patients' evolving experiences and needs. Br Med J. 2009;339.

43. Gill P, Stewart K, Treasure E, et al. Methods of data collection in qualitative research: interviews and focus groups. Br Dent J. 2008;204(6):291-5.

For peer review only - http://bmjopen.bmj.com/site/about/guidelines.xhtml

44. Office for National Statistics. 2011 Census Glossary of Terms Office for National Statistics, 2014.

45. Carter B, Coad J, Goodenough T. Community Children's Nursing in England: An appreciative review of CCNs. Department of Health Report. NHS at Home: Community Children's Nursing Services.http://www.dh.gov.uk/en/Publicationsandstatistics/Publications/PublicationsPolicyAndGuidance/DH_1248-98.: 2009.

46. Greig A, Taylor J, MacKay T. Doing Research with Children. A practical guide. London: SAGE; 2008. p. 113-20.

47. Coad J, Gibson F, Horstman M, et al. Be my guest! Challenges and practical solutions of undertaking interviews with children in the home setting. J Child Health Care. 2015;19(4):432-43.

48. Lieblich A, Tuval-Mashiach R, Zilber T. Narrative research: reading, analysis and interpretation. London: Sage; 1998.

49. Bingley A, Thomas C, Brown J, et al. Developing narrative research in supportive and palliative care: the focus on illness narratives. Palliat Med. 2008;22(5):653-8.

50. Burnard P, Gill P, Stewart K, et al. Analysing and presenting qualitative data. Br Dent J. 2008;204(8):429-32.

51. Burnard P. A method of analysing interview transcripts in qualitative research. Nurs EdToday. 1991;11:461 - 6.

52. Kendall M, Murray SA, Carduff E, et al. Use of multiperspective qualitative interviews to understand patients' and carers' beliefs, experiences, and needs. Br Med J. 2009;339.

53. Staniszewska S, Boardman F, Gunn L, et al. The Warwick Patient Experiences Framework: patient based evidence in clinical guidelines. Int J Qual Health Care. 2014;26(2):151-7.

54. Medical Research Council. MRC Ethics Guide: Medical research involving children. London. www.mrc.ac.uk: Medical Research Council. , 2004.

55. Shaw C, Brady L-M, Davey C. Guidelines for Research with Children and Young People. London: National Children's Bureau, 2011.

56. Calman L, Brunton L, Molassiotis A. Developing longitudinal qualitative designs: lessons learned and recommendations for health services research. BMC Med Res Method. 2013;13(1):1-10.

57. Hui D, De La Cruz M, Mori M, et al. Concepts and definitions for "supportive care," "best supportive care," "palliative care," and "hospice care" in the published literature, dictionaries, and textbooks. Support Care in Canc. 2013;21(3):659-85.

58. Pastrana T, Ostgarthe C, Elsner F, et al. A matter of definition - key elements identified in a discourse analysis of definitions of palliative care. Palliat Med. 2008;22:222-32.

59. Twamley K, Craig F, Kelly P, et al. Underlying barriers to referral to paediatric palliative care services: knowledge and attitudes of health care professionals in a paediatric tertiary care centre in the United Kingdom. J Child Health Care. 2014;18(1):19-30.

60. Monterosso L, Kristjanson L. Supportive and palliative care needs of families of children who die from cancer: an Australian study. Palliat Med. 2008;22(1):59-69.

61. Morstad Boldt A, Yusuf F, Himelstein BP. Perceptions of the term palliative care. J Palliat Med. 2006;9(5):1128-36.

For peer review only

62. Crocker JC, Beecham E, Kelly P et al. Inviting parents to take part in paediatric palliative care research: A mixed-methods examination of selection bias. Palliat Med. 2015;29(3):231-40.

63. Hunt A, Coad, J, West, E, et al. The Big Study for Life-limited Children and their Families: Final research report. Bristol: Together for Short Lives, 2013.

For peer review only

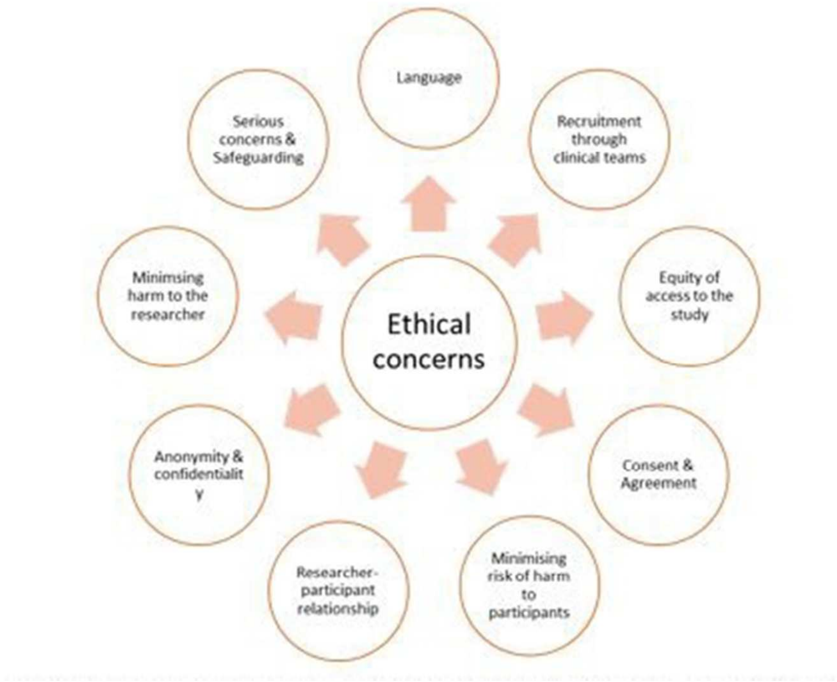

Figure 1: Ethical issues in longitudinal qualitative research for children and families in palliative care

36x31mm (300 x 300 DPI)

BMJ Editors Checklist for Qualitative Research

Checklist item	Page No
Was the research question clearly defined?	5
Overall, did the researcher make explicit in the account the theoretical framework and methods used at every stage or the research?	5,11
Was the context clearly described?	3,4,5
Was the sampling strategy clearly described and justified?<br>Was the sampling strategy theoretically comprehensive to ensure the generalisability of the conceptual analysis (diverse range of individuals and settings, for example)?	6
How was the fieldwork undertaken? Was it described in detail?	7-10
Could the evidence (fieldwork notes, interview transcripts, recordings, documentary analysis, etc) could be inspected independently by others: if relevant, could the process of transcription be independently inspected?	15, 16
Were the procedures for data analysis clearly described and theoretically justified? Did they relate to the original research questions? How were themes and concepts identified from the data?	11
Was the analysis repeated by more than one researcher to ensure reliability?	11
Did the investigator make use of quantitative evidence to test qualitative conclusions where appropriate?	N/A
Did the investigator give evidence of seeking out observations that might have contradicted or modified the analysis?	7,8,12,13,14
Was sufficient of the original evidence presented systematically in the written account to satisfy the sceptical reader of the relation between the interpretation and the evidence (for example, were quotations numbered and sources given)?	N/A

Consolidated criteria for reporting qualitative studies (COREQ): 32-item checklist

Developed from:
Tong A, Sainsbury P, Craig J. Consolidated criteria for reporting qualitative research (COREQ): a 32-item checklist for interviews and focus groups. *International Journal for Quality in Health Care*. 2007. Volume 19, Number 6: pp. 349 – 357

YOU MUST PROVIDE A RESPONSE FOR ALL ITEMS. ENTER N/A IF NOT APPLICABLE

No. Item	Guide questions/description	Reported on Page #
**Domain 1: Research team and reflexivity**		
Personal Characteristics		
1. Inter viewer/facilitator	Which author/s conducted the inter view or focus group?	1
2. Credentials	What were the researcher's credentials? E.g. PhD, MD	1
3. Occupation	What was their occupation at the time of the study?	1
4. Gender	Was the researcher male or female?	1
5. Experience and training	What experience or training did the researcher have?	8
Relationship with participants		
6. Relationship established	Was a relationship established prior to study commencement?	7
7. Participant knowledge of the interviewer	What did the participants know about the researcher? e.g. personal goals, reasons for doing the research	7
8. Interviewer characteristics	What characteristics were reported about the inter viewer/facilitator? e.g. Bias, assumptions, reasons and interests in the research topic	13-14
**Domain 2: study design**		
Theoretical framework		
9. Methodological orientation and Theory	What methodological orientation was stated to underpin the study? e.g. grounded theory, discourse analysis, ethnography, phenomenology, content analysis	6-7
Participant selection		
10. Sampling	How were participants selected? e.g. purposive, convenience, consecutive, snowball	7
11. Method of approach	How were participants approached? e.g. face-to-face, telephone, mail, email	7
12. Sample size	How many participants were in the study?	11

For peer review only

13. Non-participation	How many people refused to participate or dropped out? Reasons?	N/A
Setting		
14. Setting of data collection	Where was the data collected? e.g. home, clinic, workplace	8-9
15. Presence of non-participants	Was anyone else present besides the participants and researchers?	9
16. Description of sample	What are the important characteristics of the sample? e.g. demographic data, date	7
Data collection		
17. Interview guide	Were questions, prompts, guides provided by the authors? Was it pilot tested?	9-10
18. Repeat interviews	Were repeat inter views carried out? If yes, how many?	11
19. Audio/visual recording	Did the research use audio or visual recording to collect the data?	11
20. Field notes	Were field notes made during and/or after the inter view or focus group?	11
21. Duration	What was the duration of the inter views or focus group?	N/A
22. Data saturation	Was data saturation discussed?	11
23. Transcripts returned	Were transcripts returned to participants for comment and/or correction?	12
**Domain 3: analysis and findings**		
Data analysis		
24. Number of data coders	How many data coders coded the data?	12
25. Description of the coding tree	Did authors provide a description of the coding tree?	12
26. Derivation of themes	Were themes identified in advance or derived from the data?	12
27. Software	What software, if applicable, was used to manage the data?	12
28. Participant checking	Did participants provide feedback on the findings?	N/A
Reporting		
29. Quotations presented	Were participant quotations presented to illustrate the themes/findings? Was each quotation identified? e.g. participant number	N/A
30. Data and findings consistent	Was there consistency between the data presented and the findings?	N/A
31. Clarity of major themes	Were major themes clearly presented in the findings?	N/A
32. Clarity of minor themes	Is there a description of diverse cases or discussion of minor themes?	N/A

Once you have completed this checklist, please save a copy and upload it as part of your submission. When requested to do so as part of the upload process, please select the file type: *Checklist*. You will NOT be able to proceed with

For peer review only

submission unless the checklist has been uploaded. Please DO NOT include this checklist as part of the main manuscript document. It must be uploaded as a separate file.

For peer review only
